# Purine and carbohydrate availability drive *Enterococcus faecalis* fitness during wound and urinary tract infections

**DOI:** 10.1128/mbio.02384-23

**Published:** 2023-12-11

**Authors:** Casandra Ai Zhu Tan, Kelvin Kian Long Chong, Daryl Yu Xuan Yeong, Celine Hui Min Ng, Muhammad Hafiz Ismail, Zhei Hwee Yap, Varnica Khetrapal, Vanessa Shi Yun Tay, Daniela I. Drautz-Moses, Yusuf Ali, Swaine L. Chen, Kimberly A. Kline

**Affiliations:** 1 Singapore Centre for Environmental Life Sciences Engineering, Nanyang Technological University Singapore, Singapore, Singapore; 2 School of Biological Sciences, Nanyang Technological University Singapore, Singapore, Singapore; 3 Infectious Diseases Translational Research Programme, Division of Infectious Diseases, Department of Medicine, Yong Loo Lin School of Medicine, National University of Singapore, Singapore, Singapore; 4 Lee Kong Chian School of Medicine, Nanyang Technological University Singapore, Singapore, Singapore; 5 Singapore Eye Research Institute (SERI), Singapore General Hospital, Singapore, Singapore; 6 Laboratory of Bacterial Genomics, Genome Institute of Singapore, Singapore, Singapore; 7 Department of Microbiology and Molecular Medicine, University of Geneva, Geneva, Switzerland; University of Texas Health Science Center at Houston, Houston, Texas, USA

**Keywords:** *Enterococcus faecalis*, purines, carbohydrates, wound microenvironment, purine biosynthesis, phosphotransferase system (PTS), *in vivo *transposon sequencing, *in vivo *RNA sequencing, wound infection, fitness determinants

## Abstract

*Enterococcus faecalis* is commonly isolated from a variety of wound types. Despite its prevalence, the pathogenic mechanisms of *E. faecalis* during wound infection are poorly understood. Using a mouse wound infection model, we performed *in vivo E. faecalis* transposon sequencing and RNA sequencing to identify fitness determinants that are crucial for replication and persistence of *E. faecalis* during wound infection. We found that *E. faecalis* purine biosynthesis genes are important for bacterial replication during the early stages of wound infection, a time when purine metabolites are consumed by *E. faecalis* within wounds. We also found that the *E. faecalis* MptABCD phosphotransferase system (PTS), involved in the import of galactose and mannose, is crucial for *E. faecalis* persistence within wounds of both healthy and diabetic mice, especially when carbohydrate availability changes throughout the course of infection. During *in vitro* growth with mannose as the sole carbohydrate source, shikimate and purine biosynthesis genes were downregulated in the OG1RF ∆*mptD* mutant compared to the isogenic wild-type strain, suggesting a link between mannose transport, shikimate, and purine biosynthesis. Together, our results suggest that dynamic and temporal microenvironment changes at the wound site necessitate concomitant responses by *E. faecalis* for successful pathogenesis. Moreover, both *de novo* purine biosynthesis and the MptABCD PTS system also contribute to *E. faecalis* fitness during catheter-associated urinary tract infection, suggesting that these pathways may be central and niche-independent virulence factors of *E. faecalis* and raising the possibility of lowering exogenous purine availability and/or targeting galactose/mannose PTS to control wound infections.

**IMPORTANCE:**

Although *E. faecalis* is a common wound pathogen, its pathogenic mechanisms during wound infection are unexplored. Here, combining a mouse wound infection model with *in vivo* transposon and RNA sequencing approaches, we identified the *E. faecalis* purine biosynthetic pathway and galactose/mannose MptABCD phosphotransferase system as essential for *E. faecalis* acute replication and persistence during wound infection, respectively. The essentiality of purine biosynthesis and the MptABCD PTS is driven by the consumption of purine metabolites by *E. faecalis* during acute replication and changing carbohydrate availability during the course of wound infection. Overall, our findings reveal the importance of the wound microenvironment in *E. faecalis* wound pathogenesis and how these metabolic pathways can be targeted to better control wound infections.

## INTRODUCTION

Wound infections affect approximately 11 million people worldwide (1) and roughly USD20 billion is spent yearly on treatment (2). Wounds are broadly classified as acute or chronic. Although acute wounds heal in a predictable time course while chronic wounds are perturbed during the wound healing process(es), both are prone to colonization by a diversity of microorganisms such as enterococci (3). *Enterococcus faecalis* is a common pathogen that can colonize different wound types ranging from surgical sites to chronic ulcers and diabetic wounds (4–8). Moreover, *E. faecalis* is highly resilient to environmental stressors such as a broad pH range and high-salt conditions (9, 10), and form antibiotic tolerance-associated biofilm microcolonies on the wound bed (11, 12), together rendering *E. faecalis* wound infection difficult to treat.

Despite many studies undertaken to investigate the pathogenic mechanisms of *E. faecalis* in other infection sites, little is known regarding the pathogenicity of *E. faecalis* during wound infection. We previously showed, using a mouse wound excisional infection model, that *E. faecalis* undergoes acute replication within wounds during the first 8 h post-infection (hpi), followed by a continuous tapering of bacterial colony-forming unit (CFU) until 3 days post-infection (dpi), after which the bacterial load was maintained at 10^5^ CFU until 7 dpi (11). In the same study, *E. faecalis* multiple peptide resistance factor (MprF), which confers protection against host-derived cationic antimicrobial peptides (13–17), was identified as a fitness determinant involved in *E. faecalis* persistence in wounds at 3 dpi (11). To date, besides MprF, no other fitness (or metabolic) determinants have been identified that contribute to *E. faecalis* replication (8 hpi) and persistence (3 dpi) during wound infection.

To address this knowledge gap, we used *in vivo* transposon and RNA sequencing to probe for additional fitness determinant(s) that contribute to replication and persistence during *E. faecalis* wound infection. We identified *de novo* purine biosynthesis genes to be indispensable for *E. faecalis* acute replication during wound infection. Liquid chromatography-mass spectrometry (LC-MS) analysis of mouse wound samples showed that exogenous purine metabolites in the wound microenvironment are consumed by *E. faecalis* and thus likely insufficient to support *E. faecalis* growth during wound infection. We also identified the *E. faecalis* MptABCD phosphotransferase system (PTS) to be crucial for persistence in wounds. We characterized the *E. faecalis* MptABCD PTS as a galactose and mannose transporter and found that carbohydrate availability such as glucose, galactose, and mannose changes as wound infection progresses. In addition to a role during wound infection pathogenesis, we also showed that both *E. faecalis de novo* purine biosynthesis and MptABCD PTS contribute to *E. faecalis* fitness during catheter-associated urinary tract infection (CAUTI). Altogether, our study suggests that changes in the wound microenvironment affect *E. faecalis* pathogenesis and raise the possibility of reducing purine availability in the wound microenvironment and/or targeting MptABCD PTS as future therapeutic targets to curb chronic wound infections.

## MATERIALS AND METHODS

### Bacterial strains and growth conditions

Bacterial strains used in this study are listed in Table S1. Unless stated, all *E. faecalis* bacterial strains were grown in brain-heart infusion broth (BHI; Neogen, USA) at 37°C in static conditions for 16–18 h. Cells were harvested by centrifugation at 5,000 rpm for 5 min, and cell pellets were washed twice with 1 mL of 1× sterile phosphate-buffered saline (PBS). The final pellet was resuspended in 5 mL of 1× sterile PBS prior to optical density (OD) measurement at 600 nm. Cell suspensions were then normalized to the required cell number for the various experimental assays. When applicable, BHI were supplemented with 25 µg/mL erythromycin (Sigma-Aldrich, USA) for maintenance of pTCV and pMSP3535 plasmids.

### Mouse wound excisional model

Bacterial cultures were normalized to 2–4 × 10^8^ CFU/mL in 1× sterile PBS. Mouse wound infections were performed similar to a previous study (11). Briefly, male C57BL/6 mice (7–8 weeks old, InVivos, Singapore) or male and female *db/db* (BKS.Cg-*Dock7^m^ +/+ Lepr^db^/*J) mice (7–8 weeks or 14 weeks old, The Jackson Laboratory, USA) were anesthetized by inhalation of 3% isoflurane and the dorsal hair trimmed. A depilatory cream (Nair cream, Church and Dwight Co, USA) was then applied, and fine hair was removed through shaving with a scalpel. The skin was subsequently disinfected with 70% ethanol, and a wound was created using a 6-mm biopsy punch (Integra Miltex, USA). This was followed by inoculation with 10 µL of respective bacterial cultures per wound before the wound site was sealed with a transparent dressing (Tegaderm 3M, USA). At indicated time points, mice were euthanized, and a 1 × 1 cm piece of skin encompassing the wound site was excised and placed into 1 mL of 1× sterile PBS. Excised wounds were homogenized, and viable bacteria were enumerated by spotting onto respective selective agars. For OG1X and OG1RF selection, bacteria were spotted onto BHI solidified with 1.5% agar (Oxoid Technical No. 3) supplemented with 500 µg/mL streptomycin (MP Biomedicals, USA) or 25 µg/mL rifampicin (Sigma-Aldrich, USA), respectively. Animals that had lost the wound dressing at the time of sacrifice were excluded from data analysis. For competitive infection experiments, the competitive index (CI) was determined with the following formula:


$$CI=\frac{OG1RF_{output}/OG1X_{output}}{OG1RF_{input}/OG1X_{input}}$$

### Preparation of transposon pools for *in vivo* transposon sequencing

The *E. faecalis* transposon library containing ~15,000 mutants was constructed and kindly provided to us by Gary M. Dunny (18). Transposon mutants were arrayed into 96 wells with the transposon sequence of each mutant being known (19). Initial pools consisting of 100 transposon mutants per pool were made and glycerol stocked. Fifty transposon pools of 100 mutants were then combined to achieve a final pool size of 5,000. These pools were used for subsequent transposon sequencing, with a total of 3 distinct pools of 5,000 which covers the whole transposon library. Glycerol stocks of the 5,000 mutants were grown overnight in BHI medium (Neogen, Lansing, USA) for 15–18 h at 37°C. Overnight cultures were washed twice with 1× sterile PBS after pelleting at 5,000 rpm for 4 min and normalized to OD_600_ of 0.35 corresponding to 2 × 10^8^ CFU/mL. Wounds were made on the dorsal back of the mice as described above, and 10 µL of the normalized bacterial suspension was inoculated into wounds to achieve an infection CFU of 2 × 10^6^ CFU.

### Genomic DNA extraction for transposon sequencing

Mice were euthanized, and wounds were excised at the indicated time points. Excised wounds were subsequently homogenized in 1 mL of 1× sterile PBS. To reduce biological variance, 500 µL from two wound homogenates containing the same transposon mutants was pooled together into 4 mL of BHI for a final volume of 5 mL at 37°C for 3 h. The remaining wound homogenates were subjected to CFU enumeration on BHI agar (Acumedia, USA) supplemented with rifampicin or BHI agar without any antibiotics to check for the presence of contamination. Homogenates containing contaminants were excluded from subsequent library preparation. *In vitro* comparator pools were made by incubating 10 µL of normalized transposon overnight cultures into 4 mL of BHI broth at 37°C for 3 h as well. To recover as many transposon mutants as possible, we included an enrichment step of transposon pools for all mice samples by incubating the mixture at 37°C for 3 h, and DNA was extracted using the Qiagen DNeasy Blood and Tissue Kit (Qiagen, Germany). A total of three biological replicates of DNA samples were made from six wounds per transposon pool.

### Transposon library construction and sequencing

Extracted DNA was used for DNA library construction using NEBNext Ultra II DNA Library Prep Kit for Illumina (New England Biolabs, USA) according to the manufacturer’s instructions. DNA was subjected to acoustic shearing to obtain fragment sizes of approximately 300 bp using microtube (130 µL) (Covaris, USA). We adopted and modified the TraDIS protocol published by Barquist et al. (20) by using the proposed splinkerette design for adapter ligation and subsequent enrichment of transposon pools using an amplicon-based sequencing approach. TraDIS adapters were used for adapter ligation and PCR amplified for final library construction using the Nextera XT DNA kit (Illumina, USA) as per the manufacturer’s instructions. Constructs were normalized and sequenced as 150-bp single read using the MiSeqV3. The sequencing was done by the sequencing facility at Singapore Centre for Environmental Life Sciences Engineering (SCELSE).

### Analysis of transposon sequencing results

Reads obtained from sequencing were checked using FastQC (version 0.11.5) and adapter trimmed using bbduk from the BBMap tools (version 34.49) (21). Trimmed reads containing the 15-bp transposon sequence at the 5′ region was obtained using a customized python script and subsequently trimmed to obtain sequences for mapping. Reads were mapped onto the *E. faecalis* OG1RF (NCBI accession: GCF_000172575.2) reference genome using BWA (version 0.7.15-r1140) (22). Reads mapping to predicted open reading frames of each genome were quantified using HTSeq (23), and differential gene expression analysis was performed under the R environment (version 3.4.4) using Bioconductor package, edgeR (24). Reads were normalized based on sequencing depth, scaled for the respective library sizes using trimmed mean of M-values, with common and tagwise dispersions being estimated for downstream analysis. Genes were considered to have a fitness defect if there is a negative log2 fold change (FC), false discovery rate (FDR) ≤0.05 following correction by the Benjamin-Hochberg procedure and *P*-value ≤0.05. Gene annotation was performed using the database from the Kyoto Encyclopedia of Genes and Genomes (KEGG). Gene set enrichment analyses (GSEA) were done by comparing conditionally essential genes from 8 hpi and 3 dpi against inoculum in R using the clusterProfiler package (Version 3.16.1) (25, 26).

### RNA extraction from *in vivo* wound samples

A 1 × 1 cm of mouse skin encompassing the wound site was excised and placed into 2 mL of RNA*later* stabilization solution (Invitrogen, USA) and incubated overnight at 4°C. The mouse skin was then transferred into 1 mL of TRIzol (Life Technologies, USA) and cut into smaller pieces. The entire suspension was transferred into Lysing Matrix B 2 mL tubes (MP Biomedicals, USA) and homogenized using a FastPrep-24 tissue grinder (MP Biomedicals, USA) for 2 rounds of 40 s at 6.0 m/s with a 2 min rest on ice in between. To each sample, 200 µL of chloroform (Sigma-Aldrich, USA) was added, vortexed vigorously for 30 s and centrifuged at 12,000 × *g* for 10 min at 4°C. The top layer (aqueous phase) containing RNA was transferred to 1.5 mL tubes containing 500 µL of ice-cold ethanol and shaken vigorously before loading into the RNeasy Mini spin columns (Qiagen, Germany). Subsequent RNA extraction steps were performed according to the manufacturer’s protocol of the RNeasy Mini Kit (Qiagen, Germany). Briefly, samples were washed once with Buffer RW1, followed by two washes with Buffer RPE and elution of RNA with RNase-free water. The RNA and potential DNA contamination concentrations were quantified using Qubit RNA BR and Qubit dsDNA HS assay kits, respectively. The extracted RNA was quality checked using the RNA ScreenTape on a TapeStation instrument (Agilent Technologies, USA). Every sample had a minimum RNA concentration of 100 ng/µL, a maximum of 10% DNA contamination and a RINe value ≥8.0 before it was used for library preparation. Library preparation was done using the Ribo-Zero Plus rRNA depletion kit (Illumina, USA) to remove mouse and bacterial rRNA from the extracted total RNA samples. The RNA samples were sequenced as 75 bp paired-end reads on an Illumina HiSeq2500. Library preparation and sequencing were carried out by the SCELSE sequencing facility.

### RNA extraction from *in vitro* bacterial cultures

Overnight cultures of *E. faecalis* wild-type OG1RF and OG1RF ∆*mptD* were sub-cultured to OD_600_ of 0.01 in a 24-well microtiter plate containing 1 mL of Tryptone Soya Broth without dextrose (TSBd, Sigma-Aldrich, USA) supplemented with or without mannose or galactose (Sigma-Aldrich, USA) in biological triplicates. Bacteria were harvested at late log/early stationary phase in RNAprotect Bacteria Reagent (Qiagen, Germany) and incubated at room temperature for 5 min before centrifugation at 10,000 x *g* for 10 min. The supernatant was decanted, and bacteria pellets collected were subjected to total RNA extraction using the Qiagen RNeasy Mini Kit (Qiagen, Germany) with slight modifications. Briefly, cell pellets were resuspended in TE buffer containing 20 mg/mL lysozyme (Sigma-Aldrich, USA), further supplemented with 20 µL proteinase K (Qiagen, Germany), and incubated at 37°C for 1 h. Subsequent RNA extraction steps were performed according to the manufacturer’s protocol. The extracted RNA samples were treated with DNase (TURBO DNA-*free* kit, Invitrogen, USA) for removal of genomic DNA before it was purified using the Monarch RNA cleanup kit (New England Biolabs, USA). The RNA and potential DNA contamination concentrations were quantified using Qubit RNA BR and Qubit dsDNA HS assay kits, respectively. The extracted RNA was quality checked using the RNA ScreenTape on a TapeStation instrument (Agilent Technologies, USA) before it was sent for sequencing. Every sample had a minimum RNA concentration of 40 ng/µL, a maximum of 10% DNA contamination and a RIN value ≥8.0 before being used for library preparation and subsequent sequencing as 75 bp paired-end reads on an Illumina HiSeq2500. Similarly, library preparation and sequencing were carried out by the SCELSE sequencing facility.

### *In vivo* and *in vitro* transcriptomic analysis

All raw reads obtained were checked using FastQC (Version 0.11.9) and adaptor trimmed using bbduk from BBMap tools (Version 39.79) (21). The trimmed reads were then mapped against *E. faecalis* OG1RF (NCBI accession: CP002621) reference genome using bwa-mem of BWA (Version 0.7.17-r1188) with default options. Reads mapped to open reading frames were quantified using htseq-count of HTSeq (Version 0.12.4) with option “-m intersection-strict” (23). All rRNA counts were manually removed from all data sets. Differential gene expression analysis was performed in R using edgeR (Version 3.28.1) (24). The log2 fold change values extracted were considered significantly different based on the false discovery rate (FDR) ≤ 0.05 and *P*-value ≤ 0.05. Gene set enrichment analyses (GSEA) was done by comparing differentially expressed genes (DEGs) of different growth conditions and strains in R using the clusterProfiler package (version 3.16.1) (25, 26).

For *in vitro* transcriptome analysis, common DEGs identified between without and with carbohydrate supplementation in TSBd medium were removed, and only unique DEGs were used for downstream GSEA.

### Whole genome comparison

Genomic sequence of *E. faecalis* OG1RF (NCBI accession: CP002621) and OG1X (NCBI RefSeq assembly accession: GCF_000320305.1) were downloaded from NCBI for whole genome comparison. RagTag (version 2.1.0) (27) was used to scaffold OG1X contigs using OG1RF as the reference backbone. The largest assembled OG1X contig (~2.7 Mb) was put through Prokka (version 1.14.6) (28) for genome annotation using “--proteins” option and downloaded OG1X GenBank file from NCBI. The annotated OG1X and OG1RF (NCBI accession: CP002621) were then used for whole genome comparison using Roary (version 3.11.2) (29) to identify accessory genes between both strains.

### Molecular cloning

The primers used in this study are listed in Table S2. Transformants were screened using respective selective agar as follows: (i) *Escherichia coli* strains, LB with 500 µg/mL erythromycin (pGCP213 and pMSP3535) or 50 µg/mL kanamycin (pTCV), and (ii) *E. faecalis* strains, BHI with 25 µg/mL erythromycin (pGCP213, pMSP3535, and pTCV). The generation of *E. faecalis* deletion mutants was done by allelic replacement using pGCP213 temperature-sensitive shuttle vector described previously (30). For the construction of OG1RF ∆*purEK* and OG1RF ∆*mptD*, vector pGCP213 was linearized using *Bam*HI and *Not*I (New England Biolabs, USA). Linearized pGCP213 and inserts were ligated using In-Fusion HD Cloning Kit (Clontech, Takara, Japan) and transformed into Stellar competent cells. Successful plasmid constructs were verified by Sanger sequencing and transformed into wild-type OG1RF. Transformants were selected with erythromycin at 30°C and then passaged at non-permissive temperature at 42°C with erythromycin to select for bacteria with successful plasmid integration into the chromosome. For plasmid excision, bacteria were serially passaged at 37°C without erythromycin for erythromycin-sensitive colonies. These colonies were then subjected to PCR screening for detection of deletion mutants.

For the complementation of *purEK*, vector pTCV was linearized using *Bam*HI and *Sph*I (New England Biolabs, USA), whereas for the complementation of *mptD*, vector pMSP3535 was linearized using inverse PCR with iPCR_pMSP3535_F and iPCR_pMSP3535_R primers. Similarly, linearized pTCV and pMSP3535 as well as the respective inserts were ligated using In-Fusion HD Cloning Kit and transformed into Stellar competent cells. Successful plasmid constructs were verified by Sanger sequencing and transformed into OG1RF ∆*purEK* or OG1RF ∆*mptD*.

### Growth kinetic assay

Overnight cultures of the respective bacteria strains were diluted to OD_600_ of 0.01 in (i) RPMI 1640 medium, no phenol red (Gibco, USA) supplemented with 1% (wt/vol) casamino acids (RPMI-CA; BD Biosciences, USA) with or without inosine monophosphate (IMP); (ii) TSBd supplemented with 40 ng/mL nisin with or without 1% (wt/vol) galactose, mannose, or glucose (all purchased from Sigma-Aldrich, USA); or (iii) M9 minimal medium (Sigma-Aldrich, USA) supplemented with 1% (wt/vol) yeast extract (M9Y; Thermo Fisher Scientific, USA) with 40 ng/mL nisin with or without 1% (wt/vol) galactose, mannose, or glucose. A total of 200 µL of the diluted cell suspensions were inoculated per well in a 96-well polystyrene microtiter plate (Thermo Fisher Scientific, USA). Bacterial growth was measured at OD_600_ using a Tecan Infinite M200 Pro spectrophotometer (Tecan Group Ltd, Switzerland) every 15 min for 16 h at 37°C under static conditions.

**Fig 3:**
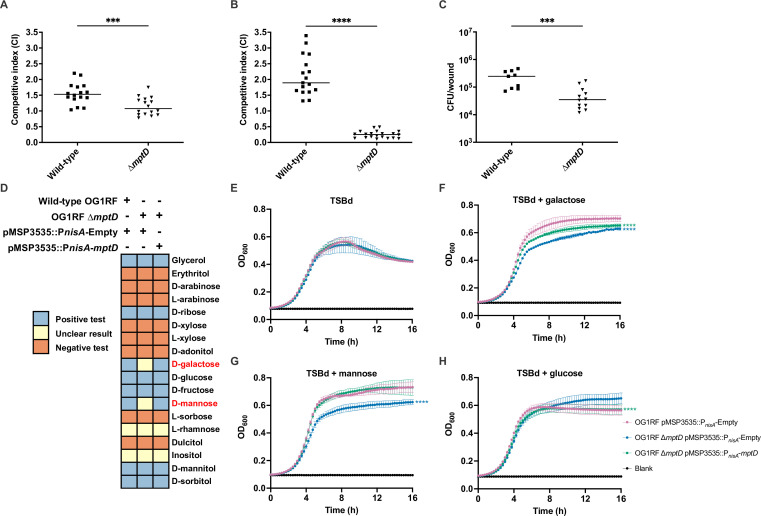
MptABCD phosphotransferase system contributes to *E. faecalis* wound fitness during persistence. Male C57BL/6 mice were wounded and infected with (**A**) a 1:1 ratio of *E. faecalis* OG1X:wild-type OG1RF or OG1X:OG1RF ∆*mptD* at 2–4 × 10^6^ CFU/wound (*N* = 3, *n* = 5–6 mice) and CFU determined at 8 hpi or (**B**) a 1:1 ratio of *E. faecalis* OG1X:wild-type OG1RF or OG1X:OG1RF ∆*mptD* at 2–4 × 10^6^ CFU/wound (*N* = 4, *n* = 5–6 mice) and CFU determined at 3 dpi or (**C**) 2–4 × 10^6^ CFU of wild-type OG1RF or OG1RF ∆*mptD* (*N* = 2, *n* = 5–6 mice) and CFU determined at 3 dpi. The recovered bacteria were enumerated on selective agar plates for each strain. Each data point represents one mouse and horizontal lines indicate the median. Statistical analysis was performed using the Mann-Whitney *U* test; ***P < 0.001, ****P < 0.0001. (**D**) Carbohydrate fermentation test (API 50 CH) of wild-type OG1RF pMPSP3535::P*_nisA_*-Empty, OG1RF ∆*mptD* pMSP3535::P*_nisA_*-Empty, and OG1RF ∆*mptD* pMSP3535::*P_nisA_-mptD*. Plasmid-based *mptD* expression was induced with 40 ng/mL nisin. Results shown are a subset of the 50 carbohydrates; differences were only detected for D-galactose and D-mannose (see Table S3 for complete table). Positive tests were determined by a change of the bromcresol purple indicator in the medium to yellow. For unclear test results, the bromcresol purple indicator did not change to yellow nor did it remain purple. Negative tests occurred when the bromcresol purple indicator remained purple. Growth kinetics of wild-type OG1RF pMPSP3535::P*_nisA_*-Empty, OG1RF ∆*mptD* pMSP3535::P*_nisA_*-Empty, and OG1RF ∆*mptD* pMSP3535::*P_nisA_-mptD* in TSBd media supplemented (**E**) without additional carbohydrates and with 1% (wt/vol) (**F**) galactose, (**G**) mannose, and (**H**) glucose over 16 h. Plasmid-based *mptD* expression was induced with 40 ng/mL nisin. Baseline readings are indicated by blank, containing only the growth media. Data are mean values of three independent biological replicates, and vertical lines represent SD from the mean. Statistical analysis was performed using generalized linear mixed model with wild-type OG1RF pMPSP3535::P*_nisA_*-Empty as the comparator; ****P < 0.001.

For growth kinetic analysis in Fig. 3E through H, the generalized linear mixed model was used for modeling, and the shape of the non-linear time-response relationship was accounted for using basis splines (B-splines). All statistical analyses were performed using R (Version 4.2.1).

### Purine metabolite quantification

Purine metabolites in mouse wounds were quantified using LC-MS performed by the Singapore Phenome Centre (SPC). A 1 × 1 cm of mouse skin encompassing the wound site was excised and placed into 1.5 mL tubes; snap frozen in liquid nitrogen; and submitted to the SPC for sample preparation, LC-MS profiling, and data processing. Briefly, tissue samples were weighed into tubes containing zirconium beads, 200 µL of 0.1 M NaOH, and 600 µL of methanol. The tubes were vortexed, homogenized, and centrifuged at 10,000 rpm for 10 min at 4°C. Two aliquots of the 300 µL supernatant were taken, dried down, and reconstituted as follows: (i) for purine quantification, 100 µL of 80:20 acetonitrile:water, 15 mM of ammonium acetate, pH 9.2, and (ii) for phosphate quantification, 100 µL of water, 20 mM of ammonium acetate, and 0.1% formic acid.

Purine and phosphate quantification was performed on a Xevo TQ-S (Waters, UK). The source temperature was set at 150°C with a cone gas flow of 150 L/h and a desolvation gas temperature of 450°C with a desolvation gas flow of 900 L/h. The capillary voltage was set to 2.5 kV in the electrospray ionization (ESI) positive or negative ionization mode for purine and phosphate quantification, respectively. For purine quantification, samples were injected into 2.1 mm × 100 mm, 1.7 µm UPLC BEH C18 column (Waters, UK) held at 45°C. Mobile phase A is water with 15 mM of ammonium acetate (pH 9.2), and mobile phase B is 90:10 acetonitrile:water with 15 mM of ammonium acetate (pH 9.2). The column flow rate was 0.4 to 0.5 mL/min. For phosphate quantification, samples were injected into 2.1 mm × 150 mm, 1.7 µm UPLC HSS T3 column (Waters, UK) held at 45°C. Mobile phase A is water with 20 mM of ammonium acetate and 0.1% formic acid. The column flow rate was 0.4 mL/min.

The weight of all tissue samples was measured prior to purine metabolite quantification, and the concentration of purine metabolites was normalized accordingly to the weight of the respective samples.

### Carbohydrate metabolism assay

Carbohydrate metabolism of *E. faecalis* strains was tested using API 50 CH (bioMérieux, France). Briefly, bacterial cultures were prepared to a turbidity equivalent of 2 McFarland standard and added to API 50 CHL Medium supplemented with 40 ng/mL nisin for induction of plasmid expression. The bacterial suspension was distributed into all 50 microtubes and sealed with mineral oil. The tray was then incubated at 37°C under static condition for 48 h and 72 h with measurements taken at each time point to monitor for variability. The results for each microtube, positive (+), negative (−) and doubtful (?), were recorded.

### Enzyme-linked immunosorbent assay (ELISA)

A 1 × 1 cm of mouse skin encompassing the wound site was first rinsed in ice-cold 1× sterile PBS and homogenized in 1 mL of ice-cold 1× sterile PBS. The homogenates then undergo two freeze-thaw cycles to break the cell membranes and centrifuged at 5,000 × *g* for 5 min at 4°C. The supernatants were collected and stored at −80°C until assessment by Mouse Glucose ELISA, Mouse Galactose ELISA, and Mannose ELISA kits (MyBioSource, USA) as per the manufacturer’s protocol. Optical density of each well was determined at OD_450_ using a Tecan Infinite M200 Pro spectrophotometer (Tecan Group Ltd., Switzerland).

### Urinary catheterization and bacterial infection

Bacterial cultures were normalized to 2–4 × 10^8^ CFU/mL in 1× sterile PBS. Implantation of catheters into mouse was performed as previously described (31). Briefly, female C57BL/6 mice (8–9 weeks old, InVivos, Singapore) were anesthetized by inhalation of 3% isoflurane. Mice were inoculated with 50 µL of bacteria suspension (~10^7^ CFU) into the urethra after catheterization. At 24 hpi, mice were euthanized. The bladders and kidneys were aseptically removed and homogenized in 1 mL and 800 µL of 1× sterile PBS, respectively. Catheters removed from the bladders were sonicated at 37 kHz and 30% power for 15 min in 1 mL of 1× sterile PBS (Elma Ultrasonic, Germany), followed by vortexing at maximum speed for another 15 min. All the samples were then serially diluted, and viable bacteria were enumerated by spotting onto the respective selective agars. For OG1X and OG1RF selection, bacteria were spotted onto BHI agar supplemented with 500 µg/mL streptomycin (MP Biomedicals, USA) or 25 µg/mL rifampicin (Sigma-Aldrich, USA), respectively, for competitive infection enumeration. Animals without catheters at the time of sacrifice were excluded from data analysis.

### Calculation of fold change

Fold change was calculated with the following formula:


$$Fold\;change=\frac{Value\;B-Value\;A}{Value\;A}$$

This formula is used to calculate the fold change of CI, purine metabolite concentrations, and carbohydrate concentrations.

### Statistical analysis

Statistical analyses were performed with GraphPad Prism software (version 9.0.0, California, USA) and are described in the respective figure legends.

## RESULTS

### *E. faecalis de novo* purine biosynthesis genes contribute to *E. faecalis* fitness during wound infection

Although *E. faecalis* is a common wound pathogen, little is known about the fitness determinants that contribute to wound infection. To identify genes contributing to fitness in wounds, using a mouse wound infection model that we have previously characterized for *E. faecalis* (11) and an *E. faecalis* OG1RF transposon mutant library consisting of ~15,000 mutants (18), we performed transposon sequencing (Tn-seq) at 8 hpi and 3 dpi following infection. Mutants disrupted in the *pur* operon (9 out of 11 genes) were significantly less abundant at 8 hpi compared to the pre-inoculation pool (Table 1; Fig. S1A). Unsurprisingly, the purine metabolism process/pathway was enriched based on the GSEA performed on significantly underrepresented genes identified from Tn-seq analysis at 8 hpi, and the only other process/pathway enriched was alanine, aspartate, and glutamate metabolism which is a pathway contributing to *E. faecalis* central metabolism (Fig. S1B). The *E. faecalis pur* operon consists of 11 genes (Fig. S1C), and *de novo* purine biosynthesis undergoes 11 reactions from L-glutamine and 5-phosphoribosyl diphosphate (PRPP) to IMP before branching into specific pathways that produce guanosine monophosphate (GMP) and adenosine monophosphate (AMP) (32) (Fig. S1D). At the same time point, we also performed *in vivo* RNA sequencing (RNA-seq) on *E. faecalis* wild-type OG1RF from infected wounds to provide a genome-wide analysis of differential gene expression during wound infection. We predicted that genes identified as essential for wound fitness using Tn-seq may display an increased gene expression, yielding a correlation coefficient between Tn-seq and RNA-seq close to −1. However, the calculated Spearman rank correlation coefficient between fold change of mutant abundance and fold change of differential expression among statistically significant genes was −0.0311 (Fig. S2A), which was similar to a previous study looking at fitness determinants and gene expression during *P. aeruginosa* wound infection (33). Nevertheless, our observation that all 11 genes in the *pur* operon were significantly upregulated at 8 hpi compared to inoculum (Table 2; Fig. S2B), suggested a role for purine biosynthesis during acute *E. faecalis* wound infection. Among the other enriched processes/pathways identified from GSEA based on significantly differentially expressed genes between *E. faecalis* wild-type OG1RF inoculum and wild-type OG1RF-infected wounds, the genes in processes/pathways such as β-lactam resistance, peptidoglycan biosynthesis, quorum sensing, fatty acid metabolism, fatty acid biosynthesis, and ABC transporters were significantly upregulated in the infected wounds (Fig. S2B). By contrast, the genes in processes/pathways such as lysine biosynthesis; microbial metabolism in diverse environments; valine, leucine, and isoleucine degradation; glycerolipid metabolism; propanoate metabolism; galactose metabolism; amino sugar and nucleotide sugar metabolism; starch and sucrose metabolism; fructose and mannose metabolism; and phosphotransferase system were significantly downregulated in the infected wounds (Fig. S2B). These changes in processes/pathways show that *E. faecalis* undergoes significant transcriptional adaptation within the first 8 hpi.

**TABLE 1:** *E. faecalis* transposon mutant abundance profiled by Tn-seq from 8 hpi wounds^*a*^

Locus tag	Name	Description	Log_2_ FC	*P*-value	FDR
OG1RF_10019	*mptAB*	PTS mannose transporter subunit EIIAB	−1.15	5.75E-06	2.11E-05
OG1RF_10020	*mptC*	PTS mannose/fructose/sorbose transporter subunit IIC	−0.92	3.54E-10	1.58E-09
OG1RF_10021	*mptD*	PTS mannose transporter subunit IID	−1.11	8.90E-18	7.46E-17
OG1RF_11489	*purD*	Phosphoribosylamine–glycine ligase	−1.70	1.12E-06	3.48E-06
OG1RF_11490	*purH*	Inosine monophosphate cyclohydrolase	−2.29	1.61E-37	5.62E-36
OG1RF_11491	*purN*	Phosphoribosylglycinamide formyltransferase	−1.16	1.63E-02	3.03E-02
OG1RF_11492	*purM*	Phosphoribosylformylglycinamidine cyclo-ligase	−2.06	1.87E-15	1.30E-14
OG1RF_11493	*purF*	Amidophosphoribosyltransferase	−3.19	3.23E-27	1.82E-26
OG1RF_11494	*purL*	Phosphoribosylformylglycinamidine synthase II	−1.79	1.38E-11	3.68E-11
OG1RF_11495	*purL2*	Phosphoribosylformylglycinamidine synthase subunit PurQ	−3.03	2.91E-37	2.59E-36
OG1RF_11496	*purS*	Phosphoribosylformylglycinamidine synthase	−2.68	7.75E-15	5.12E-14
OG1RF_11498	*purK*	5-(Carboxyamino)imidazole ribonucleotide synthase	−2.15	9.76E-13	5.37E-12

^*a*^ Complete table can be found in Supplementary file 1 (sheet 1).

**TABLE 2:** *E. faecalis* purine biosynthesis genes differentially regulated from 8 hpi wounds^*a*^

Locus tag	Name	Description	Log_2_ FC	*P*-value	FDR
OG1RF_11489	*purD*	Phosphoribosylamine–glycine ligase	6.47	5.40E-199	4.36E-197
OG1RF_11490	*purH*	Inosine monophosphate cyclohydrolase	6.26	2.78E-182	1.50E-180
OG1RF_11491	*purN*	Phosphoribosylglycinamide formyltransferase	6.27	1.91E-115	3.48E-114
OG1RF_11492	*purM*	Phosphoribosylformylglycinamidine cyclo-ligase	7.02	7.40E-132	1.73E-130
OG1RF_11493	*purF*	Amidophosphoribosyltransferase	7.40	1.57E-141	4.15E-140
OG1RF_11494	*purL*	Phosphoribosylformylglycinamidine synthase II	6.58	9.95E-152	3.10E-150
OG1RF_11495	*purL2*	Phosphoribosylformylglycinamidine synthase subunit PurQ	6.12	1.16E-82	1.35E-81
OG1RF_11496	*purS*	Phosphoribosylformylglycinamidine synthase	6.35	7.15E-22	2.25E-21
OG1RF_11497	*purC*	Phosphoribosylaminoimidazolesuccinocarboxamide synthase	7.29	6.75E-118	1.28E-116
OG1RF_11498	*purK*	5-(Carboxyamino)imidazole ribonucleotide synthase	5.37	1.57E-62	1.20E-61
OG1RF_11499	*purE*	5-(Carboxyamino)imidazole ribonucleotide mutase	5.24	1.24E-75	1.29E-74

^*a*^ Complete table can be found in Supplementary file 1 (sheet 2).

**Fig 1:**
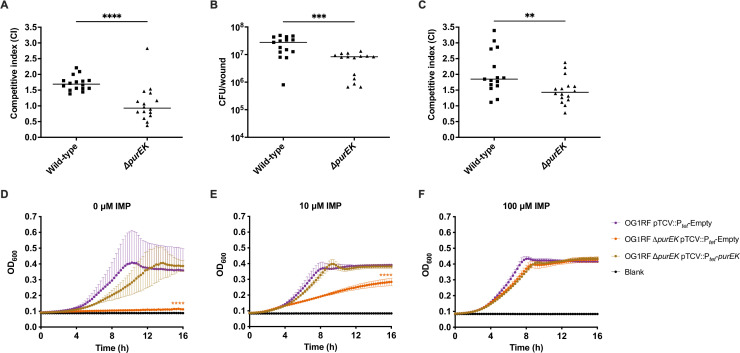
*De novo* purine biosynthesis contributes to *E. faecalis* fitness during early stages of wound infection. Male C57BL/6 mice were wounded and infected with (**A**) a 1:1 ratio of *E. faecalis* OG1X:wild-type OG1RF or OG1X:OG1RF ∆*purEK* at 2–4 × 10^6^ CFU per wound, (**B**) 2–4 × 10^6^ CFU of wild-type OG1RF or OG1RF ∆*purEK* in single-strain infection and CFU determined at 8 hpi, or (**C**) a 1:1 ratio of *E. faecalis* OG1X:wild-type OG1RF or OG1X:OG1RF ∆*purEK* at 2–4 × 10^6^ CFU per wound and CFU determined at 3 dpi. The recovered bacteria were enumerated on selective agar plates for each strain. Each data point represents one mouse, and horizontal lines indicate the median, *N* = 3, *n* = 5–6 mice per group per experiment. Statistical analysis was performed using the Mann-Whitney *U* test; **P < 0.01, ***P < 0.001, ****P < 0.0001. Growth kinetics of wild-type OG1RF pTCV::P*_tet_*-Empty, OG1RF ∆*purEK* pTCV::P*_tet_*-Empty, and OG1RF ∆*purEK* pTCV::P*_tet_-purEK* in RPMI-CA media supplemented with (**D**) 0, (**E**) 1, or (**F**) 100 µM IMP over 16 h. Baseline readings are indicated by blank, containing only the growth media. Data are mean values of three independent biological replicates, and vertical lines represent SD from the mean. Statistical analysis was performed at 16 h OD_600_ measurement with wild-type OG1RF pTCV::P*_tet_*-Empty as the comparator using the Mann-Whitney *U* test; ****P < 0.0001.

To confirm the role of *de novo* purine biosynthesis for *E. faecalis* replication during wound infection, we created an in-frame deletion mutant of the first two genes in the *pur* operon, *purEK* (OG1RF ∆*purEK*) (34), and performed an *in vivo* competitive infection with *E. faecalis* OG1X at 8 hpi, a closely related strain expressing different antibiotic resistance genes enabling differential selection (35). In accordance with our Tn-seq and RNA-seq results, OG1RF ∆*purEK* (CI = 0.93) displayed a statistically significant reduction in fitness (0.45-fold decrease) compared to wild-type OG1RF (CI = 1.69) (Fig. 1A). We subsequently performed a single-strain infection and observed that OG1RF ∆*purEK* CFUs were lower than wild-type OG1RF (8.33 × 10^6^ vs 2.77 × 10^7^ CFU per wound; 0.70-fold decrease) in a statistically significant manner at 8 hpi (Fig. 1B). We performed a whole genome comparison of *E. faecalis* OG1RF and OG1X to determine whether single-nucleotide polymorphisms between the two strains could contribute to changes in fitness during the *in vivo* competitive infection. The two strains share a core set of 2,486 genes, and the differences in accessory genes predominantly encode hypothetical proteins (File S1; sheet 3). While we cannot rule out the possibility that some of the differences between these two strains also contribute to infection fitness, the fact that single-strain infections (where OG1X is absent) display fitness defects for the *purEK* mutant demonstrate that the changes in wound fitness are directly attributable to *purEK* (Fig. 1B).

Tn-seq analysis at 3 dpi also suggested the importance of *de novo* purine biosynthesis for persistence in wounds, as two genes in the *pur* operon (*purH* and *purM*) were significantly less abundant in the post-infection transposon pools (Table 3). To validate the contribution of purine biosynthesis at 3 dpi, we similarly performed the *in vivo* competitive infection with OG1X. Although OG1RF ∆*purEK* (CI = 1.43) displayed a statistically significant reduction in fitness (0.23-fold decrease) compared to wild-type OG1RF (CI = 1.85) at 3 dpi (Fig. 1C), the difference in CI was not as large as compared to 8 hpi (Fig. 1A). Overall, these results demonstrate that *E. faecalis de novo* purine biosynthesis contributes to *E. faecalis* replication during acute infection as well as to persistence in wounds.

**TABLE 3:** *E. faecalis* transposon mutant abundance profiled by Tn-seq from 3 dpi wounds^*a*^

Locus tag	Name	Description	Log_2_ FC	*P*-value	FDR
OG1RF_10019	*mptAB*	PTS mannose transporter subunit EIIAB	−9.75	5.64E-05	2.93E-03
OG1RF_10020	*mptC*	PTS mannose/fructose/sorbose transporter subunit IIC	−10.36	3.81E-06	8.53E-05
OG1RF_10021	*mptD*	PTS mannose transporter subunit IID	−10.86	1.10E-08	6.29E-07
OG1RF_11280	*aroE*	Shikimate dehydrogenase	−9.76	7.37E-05	3.34E-03
OG1RF_11281	*aroF*	3-Deoxy-7-phosphoheptulonate synthase	−9.20	2.70E-03	1.60E-02
OG1RF_11490	*purH*	Inosine monophosphate cyclohydrolase	−10.30	2.52E-06	6.12E-05
OG1RF_11492	*purM*	Phosphoribosylformylglycinamidine cyclo-ligase	−9.01	4.66E-03	2.42E-02

^*a*^ Complete table can be found in Supplementary file 1 (sheet 4).

We next validated the predicted requirement of *de novo* purine biosynthesis for *E. faecalis* growth in RPMI-CA medium lacking purines. As expected, the deletion of *purEK* (OG1RF ∆*purEK* pTCV::P*_tet_*-Empty) resulted in severe growth attenuation compared to wild-type (OG1RF pTCV::P*_tet_*-Empty), and complementation of *purEK* on a plasmid (OG1RF ∆*purEK* pTCV::P*_tet_-purEK*) restored growth to near wild-type levels (Fig. 1D). To confirm that the disruption of the purine biosynthesis pathway was the sole reason for the growth attenuation observed, we supplemented the RPMI-CA medium with 10 and 100 µM IMP (end-product of purine biosynthesis; Fig. S1D). With supplementation of IMP, the growth of OG1RF ∆*purEK* pTCV::P*_tet_*-Empty was restored to wild-type levels in a dose-dependent manner (Fig. 1E and F). These findings demonstrate the importance of the *pur* operon for *E. faecalis* growth in a purine-deficient environment.

### Purine metabolites in wounds are low during the acute replication phase of *E. faecalis* infection

**Fig 2:**
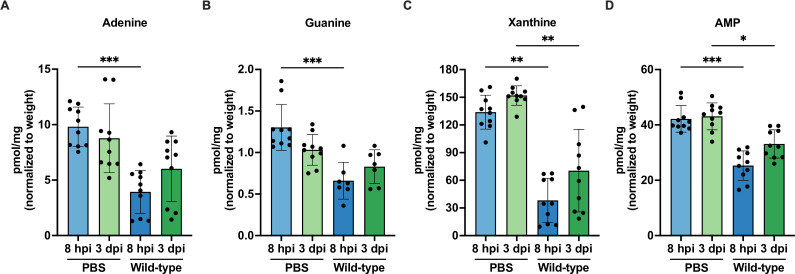
Purine metabolites are low during early *E. faecalis* wound infection. Male C57BL/6 mice were wounded and inoculated with PBS or 2–4 × 10^6^ CFU of wild-type OG1RF. Wounds were harvested at 8 hpi and 3 dpi for quantification of (**A**) adenine, (**B**) guanine, (**C**) xanthine, and (**D**) AMP using LC-MS. Each data point represents one mouse, and error bars represent SD from the mean; *N* = 2, *n* = 5 mice per group per experiment. Statistical analysis was performed using the Mann-Whitney *U* test; *P < 0.05, **P < 0.01, ***P < 0.001.

*De novo* purine biosynthesis is required for *Staphylococcus aureus* pathogenesis during bacteremia because the disruption of purine biosynthesis leads to reduced bacterial virulence as measured by animal weight loss and bacterial burden (36). Moreover, previous Tn-seq studies revealed that purines were among the metabolites deemed “not available” to *P. aeruginosa* during both burn and chronic wound infections (33). Collectively, these studies demonstrate the significance of purine biosynthesis for virulence across various bacteria and infection sites. Hence, we predicted that purine availability in the wound microenvironment is low, which would explain the importance of *de novo* purine biosynthesis for successful *E. faecalis* wound infection. To assess whether purines were indeed limited during wound infection, we quantified purine metabolites from *E. faecalis* wild-type OG1RF-infected wounds compared to PBS-treated control wounds at 8 hpi and 3 dpi using LC-MS. Following infection with *E. faecalis* wild-type, we observed a trend toward decreased purine metabolites compared to PBS-treated wounds at 8 hpi which was statistically significant for adenine (0.60-fold decrease), guanine (0.49-fold decrease), xanthine (0.71-fold decrease), and AMP (0.40-fold decrease) (Fig. 2A through D, compare blue bars), suggesting that there may be consumption of purine metabolites during acute wound infection which could explain the high demand for purine metabolites when *E. faecalis* is replicating in the wounds. By contrast, the levels of purine metabolites remain similar between PBS-treated and wild-type OG1RF-infected wounds at 3 dpi, except for xanthine (0.54-fold decrease) and AMP (0.23-fold decrease) (Fig. 2C and D, compare green bars). Differences following infection were not observed for adenosine, guanosine, and inosine (Fig. S3A through C). Additionally, there were no significant differences in purine metabolite concentrations between PBS-treated wounds at 8 hpi and 3 dpi (Fig. 2), suggesting that the decrease in purines was likely not host driven but was due to *E. faecalis* consumption.

As purine biosynthesis is disrupted in OG1RF ∆*purEK* and the strain would likely depend on exogenous purines in the wound microenvironment as a source for purines during wound infection (i.e., consuming more purines from the wound microenvironment), we hypothesized that there would be a further decrease in purine metabolite levels when mouse wounds are infected with OG1RF ∆*purEK* compared to wild-type OG1RF-infected wounds at 8 hpi. Therefore, we quantified purine metabolites from PBS-treated control wounds, *E. faecalis* wild-type OG1RF-, and OG1RF ∆*purEK*-infected wounds at 8 hpi using LC-MS. However, the levels of purine metabolites (adenine, xanthine, adenosine, guanosine, and inosine) remained similar between wild-type OG1RF- and OG1RF ∆*purEK*-infected wounds (Fig. S3D through H), suggesting that there is no detectable increase in consumption of purine metabolites by OG1RF ∆*purEK* during acute wound infection. Nonetheless, our results suggest that the importance of *de novo* purine biosynthesis for *E. faecalis* replication during acute wound infection is likely driven by its consumption of purine metabolites in the wound microenvironment.

### *E. faecalis* MptABCD phosphotransferase system is important for *E. faecalis* persistence during wound infection

Tn-seq analysis at 8 hpi and 3 dpi also identified *mptABCD* as significantly contributing to *E. faecalis* fitness at both time points (Tables 1 and 3). Similar to 8 hpi, we performed GSEA on significantly underrepresented genes identified from Tn-seq analysis at 3 dpi, but we did not observe any enriched processes/pathways. To identify any unique enriched processes/pathways between 8 hpi and 3 dpi, we compared the significantly underrepresented genes identified from Tn-seq analysis at both 8 hpi and 3 dpi and performed a GSEA on the unique genes at both timepoints. However, the GSEA performed on unique genes at 8 hpi did not reveal any other processes/pathways that were not already identified (Fig. S1B and S4A), and there were no processes/pathways enriched at 3 dpi. We, therefore, investigated the contribution of *mptABCD* to *E. faecalis* virulence during wound infection, especially at 3 dpi when transposon insertions in all genes of the *mpt* operon were among the most significantly underrepresented following Tn-seq (Fig. S4B). *E. faecalis mptABCD* encodes a carbohydrate-specific phosphotransferase system used for the import of carbohydrates (37) to facilitate wound persistence. Based on the KEGG genome database, *E. faecalis mptABCD* is predicted to encode PTS mannose/fructose/sorbose transporter subunits.

To validate the role of the *E. faecalis* MptABCD PTS during wound infection, we created an in-frame deletion mutant of *mptD* (OG1RF ∆*mptD*) and performed an *in vivo* competitive infection with OG1X at 8 hpi and 3 dpi. In agreement with our Tn-seq results, OG1RF ∆*mptD* had a statistically significant reduction in fitness compared to wild-type OG1RF at both 8 hpi and 3 dpi (Fig. 3A and B). However, we observed a bigger difference in CI at 3 dpi (CI_OG1RF_ = 1.89, CI_∆*mptD*_ = 0.23; 0.88-fold decrease) compared to 8 hpi (CI_OG1RF_ = 1.53, CI_∆*mptD*_ = 1.08; 0.30-fold decrease), which may explain the more pronounced decrease in log_2_ FC that we observed in the post-infection transposon pools at 3 dpi (Tables 1 and 3). Similarly, we performed a single-strain infection and observed that OG1RF ∆*mptD* colonized more poorly than wild-type OG1RF (3.50 × 10^4^ vs 2.47 × 10^5^ CFU per wound; 0.86-fold decrease) in a statistically significant manner at 3 dpi (Fig. 3C). Taken together, these results indicate that MptABCD PTS plays a role during *E. faecalis* persistence in wounds.

### *E. faecalis* MptABCD phosphotransferase system is responsible for the import of galactose and mannose

To determine the carbohydrate(s) transported by the MptABCD PTS, we tested the ability of *E. faecalis* to metabolize 50 different carbohydrates. Across all 50 carbohydrates, the deletion of *mptD* (OG1RF ∆*mptD* pMSP3535::P*_nisA_*-Empty) only affected the metabolism of galactose and mannose when compared to wild-type (OG1RF pMSP3535::P*_nisA_*-Empty), and complementation of *mptD* on an inducible plasmid (OG1RF ∆*mptD* pMSP3535::P*_nisA_-mptD*) restored the mannose and galactose metabolism (Fig. 3D). To validate that the *mptD* deletion mutant was indeed unable to metabolize galactose and mannose, we performed growth kinetic assays of wild-type OG1RF, *mptD* deletion, and complement strains in TSBd and M9Y [M9 minimal medium with 1% (wt/vol) yeast extract] growth medium supplemented with different carbohydrates. We did not observe any growth differences between all three strains in TSBd and M9Y in the absence of carbohydrate supplementation (Fig. 3E; Fig. S5A). When TSBd and M9Y was supplemented with either galactose or mannose, the growth of OG1RF pMSP3535::P*_nisA_*-Empty was augmented, but OG1RF ∆*mptD* pMSP3535::P*_nisA_*-Empty growth was not (Fig. 3F and G; Fig. S5B and C). Complementation of *mptD* in the deletion mutant resulted in improved growth compared to OG1RF ∆*mptD*pMSP3535::P*_nisA_*-Empty levels when TSBd was supplemented with galactose (Fig. 3F) and was almost identical to wild-type OG1RF pMSP3535::P*_nisA_*-Empty levels with mannose supplementation (Fig. 3G). Even though complementation of *mptD* resulted in improved growth compared to wild-type OG1RF pMSP3535::P*_nisA_*-Empty levels when M9Y was supplemented with galactose and mannose, it did not improve the growth to wild-type OG1RF pMSP3535::P*_nisA_*-Empty levels (Fig. S5B and C). As a control, we also supplemented TSBd and M9Y with glucose, for which we did not expect to observe any growth differences between the three strains as metabolism of glucose was unaffected when *mptD* was deleted (Fig. 3D). Although growth differences were similar between all strains, at least through log phase, when TSBd was supplemented with glucose (Fig. 3H), the growth of OG1RF ∆*mptD* pMSP3535::P*_nisA_*-Empty was augmented compared to wild-type OG1RF pMSP3535::P*_nisA_*-Empty and OG1RF ∆*mptD* pMSP3535::P*_nisA_-mptD* in the stationary phase (Fig. 3H). By contrast, growth differences were similar between all strains when M9Y was supplemented with glucose (Fig. S5D). Collectively, these results indicate that MptABCD PTS is responsible for the import for galactose and mannose into *E. faecalis* and that the import of these carbohydrates may contribute to *E. faecalis* persistence in wounds.

### Carbohydrate availability changes as the wound infection progresses

**Fig 4:**
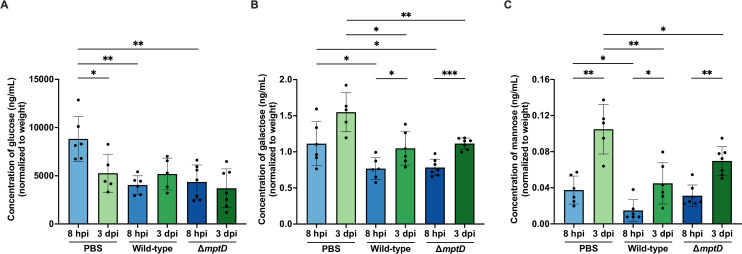
Carbohydrate availability changes as *E. faecalis* wound infection progresses. Male C57BL/6 mice were wounded and inoculated with sterile PBS, wild-type OG1RF, or OG1RF ∆*mptD* at 2–4 × 10^6^ CFU per wound. Wounds were harvested at 8 hpi and 3 dpi and subjected to (**A**) glucose, (**B**) galactose, and (**C**) mannose quantification by ELISA. Each data point represents measurement from one mouse; error bars represent SD from the mean; *N* = 1, *n* = 6–7 mice. Statistical analysis was performed using the Mann-Whitney *U* test; *P < 0.05, **P < 0.01, ***P < 0.001.

Galactose and mannose imports appear to be more crucial at 3 dpi than at 8 hpi (Fig. 3A and B). We reasoned that carbohydrate availability in wounds may change as wound infection progresses, where successful *E. faecalis* persistence is dependent on its promiscuous ability to source from a wide array of nutrients in the wound microenvironment. We hypothesized that these changes in carbohydrate availability influence the natural course of wound pathogenesis, and *E. faecalis* depletion of preferred carbohydrate sources such as glucose during acute infection would necessitate a switch to other carbohydrates such as mannose and galactose at later time points. To test this hypothesis, we harvested PBS-treated, *E. faecalis* wild-type OG1RF- and OG1RF ∆*mptD*-infected wounds at 8 hpi and 3 dpi and quantified the concentrations of glucose, galactose, and mannose. Consistent with our prediction, we detected lower glucose concentrations (0.40-fold decrease) at 3 dpi compared to 8 hpi in PBS-treated wounds (Fig. 4A) and concordant higher galactose (0.39-fold increase) and mannose (1.80-fold increase) at 3 dpi compared to 8 hpi (Fig. 4B and C). Additionally, *E. faecalis* wild-type OG1RF infection further decreased glucose (0.54-fold decrease), galactose (0.31-fold decrease), and mannose (0.60-fold decrease) concentrations compared to PBS-treated wounds at 8 hpi (Fig. 4A through 4C), suggesting that *E. faecalis* can use all three carbohydrates during early phases of wound infection, which may support its growth. At 3 dpi, however, we detected no significant differences in glucose concentrations in any infected wounds (Fig. 4A) and observed that *E. faecalis* wild-type OG1RF- (0.32-fold decrease) and OG1RF ∆*mptD*-infected (0.28-fold decrease) wounds had lesser galactose than PBS-treated wounds (Fig. 4B). Likewise, lesser mannose (0.57-fold decrease) was detected compared to PBS-treated wounds when infected with *E. faecalis* wild-type OG1RF at 3 dpi (Fig. 4C). These results indicate that *E. faecalis* can import and deplete galactose and mannose availability during wound infection. Consistent with this conclusion, mannose concentrations were similar between PBS-treated and OG1RF ∆*mptD*-infected wounds at 8 hpi and higher in OG1RF ∆*mptD*-infected wounds compared to OG1RF-infected wounds at 3 dpi (0.55-fold increase; albeit not significant) (Fig. 4C), suggesting that disruption of MptABCD PTS indeed leads to decreased mannose import (i.e., mannose accumulation) *in vivo*. Unexpectedly, we did not detect statistically significant differences in galactose concentration between wild-type OG1RF- and OG1RF ∆*mptD*-infected wounds at any time point (Fig. 4B), despite a role for MptABCD in galactose metabolism *in vitro* (Fig. 3D). Since we observed minimal glucose depletion by *E. faecalis* at 3 dpi, we wondered whether a high glucose microenvironment (such as that found during hyperglycemia in diabetic mice) would prompt *E. faecalis* to import glucose instead of galactose and mannose and consequently render the MptABCD PTS dispensable during persistence in diabetic animals. As such, we performed an *in vivo* competitive infection of OG1RF ∆*mptD* with OG1X in diabetic (*db/db*) mice at 3 dpi. However, like non-diabetic mice, OG1RF ∆*mptD* (CI = 0.34) had a statistically significant reduction in fitness (0.72-fold decrease) compared to wild-type OG1RF (CI = 1.22) at 3 dpi (Fig. S5E). Taken together, these results suggest that as the wound infection progresses, glucose becomes depleted and other carbohydrates, such as galactose and mannose, become more available in the wounds. As such, *E. faecalis* undergoes a metabolic switch toward galactose and mannose metabolism as the wound infection progresses.

### *E. faecalis de novo* purine and shikimate biosyntheses are regulated by MptABCD phosphotransferase system-mediated mannose import

**Fig 5:**
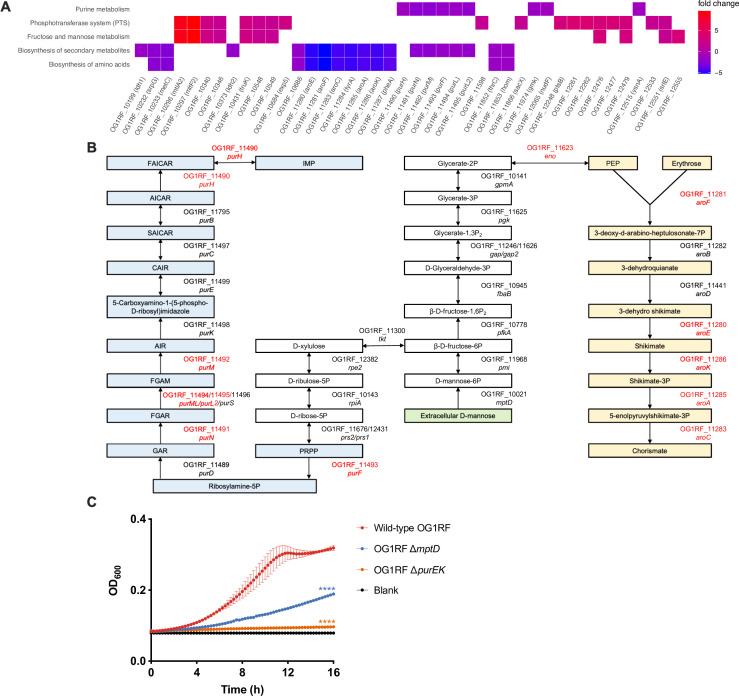
Mannose imported by *E. faecalis mptABCD* is functionally linked to *de novo* purine and shikimate biosyntheses. (**A**) Gene set enrichment pathways identified based on differentially expressed genes between wild-type OG1RF and OG1RF ∆*mptD* grown in TSBd supplemented with 1% (wt/vol) mannose. Complete table of differentially expressed genes can be found in online supplementary file 1 (sheets 6–10). (**B**) KEGG pathways depicting how import of extracellular D-mannose by MptD (green) acts as a substrate for *de novo* purine (blue) and shikimate (yellow) biosyntheses. (**C**) Growth kinetics of wild-type OG1RF, OG1RF ∆*mptD*, and OG1RF ∆*purEK* in RPMI-CA over 16 h. Baseline readings are indicated by blank, containing only the growth media. Data are mean values of three independent biological replicates, and vertical lines represent SD from the mean. Statistical analysis was performed at 16 h OD_600_ measurement with wild-type OG1RF as the comparator using the Mann-Whitney *U* test; ****P < 0.0001.

Since MptABCD PTS imports galactose and mannose and this contributes to *E. faecalis* virulence *in vivo*, we asked whether there were any galactose and/or mannose-dependent changes in gene expression that might further explain why, in particular, these carbohydrates are important during wound infection. We, therefore, performed *in vitro* RNA-seq with wild-type OG1RF and OG1RF ∆*mptD* grown in TSBd without and with supplementation of galactose or mannose. Although there were differentially expressed genes observed upon the supplementation of galactose (online supplementary file 1; sheet 5), we did not observe any enriched processes/pathways based on GSEA. By contrast, when wild-type OG1RF and OG1RF ∆*mptD* were grown in TSBd supplemented with mannose, we observed several processes/pathways such as purine metabolism, PTS, fructose and mannose metabolism, and biosynthesis of secondary metabolites and amino acids that were enriched (Fig. 5A). Among the enriched processes/pathways, 7 out of 8 genes in the shikimate biosynthesis operon (*aroF*, *aroE*, *aroC*, *tyrA*, *aroA*, *aroK*, and *pheA*) and 6 out of 11 genes in the *pur* operon (*purH*, *purN*, *purM*, *purF*, *purL*, and *purL2*) were significantly downregulated in OG1RF ∆*mptD* when grown in TSBd supplemented with mannose (Fig. 5A), suggesting that shikimate and purine biosyntheses are attenuated when import of mannose is hindered. KEGG pathway analysis showed that mannose imported by MptABCD PTS was functionally linked to shikimate and purine biosyntheses (Fig. 5B). Following mannose import by MptABCD PTS, it can undergo a series of reactions that lead to the production of PEP (a substrate of shikimate pathway) or production of PRPP (a substrate for purine biosynthesis) (Fig. 5B). These results were also supported by Tn-seq analysis whereby transposon mutants for some genes of the shikimate biosynthesis and *pur* operon were significantly underrepresented in the post-infection transposon pools at 3 dpi (Table 3).

To subsequently validate that purine biosynthesis was impeded when mannose import was hindered in OG1RF ∆*mptD*, we performed a growth kinetic assay in RPMI-CA medium lacking purines. In agreement with our *in vitro* RNA-seq analysis, the growth of OG1RF ∆*mptD* was attenuated compared to wild-type OG1RF in RPMI-CA medium (Fig. 5C). These results confirm that hindered mannose import by MptABCD PTS also reduces *de novo* purine biosynthesis. To summarize, at 8 hpi, the infected wound microenvironment has lower levels of purine metabolites presumably due to consumption by *E. faecalis* (Fig. 2) and high levels of mannose (Fig. 4C). We found that purine availability is important to achieve high titers during the onset of *E. faecalis* wound infection. Therefore, our data suggest that *E. faecalis* lacking *mptD* would be attenuated due to its inability to overcome the low purine bioavailability by being unable to uptake mannose to trigger its own purine biosynthesis.

### *E. faecalis de novo* purine biosynthesis and MptABCD phosphotransferase system are important for catheter-associated urinary tract infection

**Fig 6:**
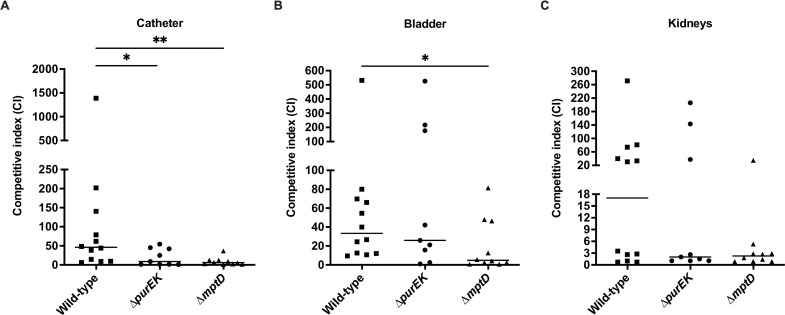
*De novo* purine biosynthesis and the MptABCD phosphotransferase system contribute to *E. faecalis* fitness during CAUTI. Female C57BL/6 mice were implanted with 5-mm silicon catheters in the bladders and infected with a 1:1 ratio of 10^7^ CFU of *E. faecalis* OG1X:wild-type OG1RF, OG1X:OG1RF ∆*purEK,* or OG1X:OG1RF ∆*mptD*. (**A**) Catheters, (**B**) bladders, and (**C**) kidneys were harvested at 24 hpi, and the recovered bacteria were enumerated on selective agar plates for each strain. Each data point represents one mouse, and horizontal lines indicate the median; *N* = 2, *n* = 6 mice per group per experiment. Statistical analysis was performed using the Mann-Whitney *U* test; *P < 0.05, **P < 0.01.

Purine biosynthesis is essential in a variety of infection types (36, 38–42). Additionally, purines are thought to be limited in the urinary tract since an *E. coli guaA* mutant (defective in guanine biosynthesis) was significantly less virulent than its parental wild type in a urinary tract infection (UTI) mouse model (43), we, therefore, wondered whether *E. faecalis de novo* purine biosynthesis and MptABCD PTS would similarly contribute to other *E. faecalis* infections. In addition to being a common wound pathogen, *E. faecalis* is also a frequently isolated uropathogen (44). Hence, we tested the contribution of purine biosynthesis and MptABCD PTS in a CAUTI model, assessing *in vivo* competitive infections of OG1RF ∆*purEK* and OG1RF ∆*mptD* with OG1X at 1 dpi. We observed that OG1RF ∆*purEK* (CI = 8.99; 0.81-fold decrease) and OG1RF ∆*mptD* (CI = 6.01; 0.87-fold decrease) had a statistically significant reduction in fitness compared to wild-type OG1RF (CI = 46.19) on catheters (Fig. 6A), while only OG1RF ∆*mptD* (CI = 4.83) had a statistically significant reduction in fitness (0.86-fold decrease) compared to wild-type OG1RF (CI = 33.33) in the bladder (Fig. 6B), and neither *purEK* nor *mptD* contributed to fitness in the kidneys (Fig. 6C). These results suggest that *de novo* purine biosynthesis and the MptABCD PTS may be central and niche-independent virulence factors of *E. faecalis*.

## DISCUSSION

In this study, we sought to identify fitness determinants that are crucial for acute *E. faecalis* replication and later persistence in wounds. We show that both *E. faecalis de novo* purine biosynthesis and the MptABCD PTS are important for *E. faecalis* acute replication and persistence, respectively. We report that purine metabolites are lower at the wound site, likely due to consumption by *E. faecalis* during the early stages of wound infection compared to later persistent stages, explaining the importance of *de novo* purine biosynthesis for acute *E. faecalis* wound infection. We also show that carbohydrate availability in the wound microenvironment has more galactose and mannose as the wound infection progresses, providing a reason for the requirement of the MptABCD galactose and mannose transporter during persistent *E. faecalis* wound infection.

Nucleotides play a critical role in cell physiology of both prokaryotes and eukaryotes, such as DNA and RNA synthesis, enzyme cofactors (NAD^+^ and FAD^+^), and energy carriers (ATP and GTP), and are also involved in the biosynthesis of riboflavin (45, 46). *De novo* purine biosynthesis is required for many pathogens to establish a successful infection. For example, purine biosynthesis is necessary for successful proliferation of Gram-negative *E. coli* and *Salmonella typhimurium* in human serum (42) and *P. aeruginosa* in wounds (33) as well as for Gram-positive *Streptococcus pyogenes* growth in human blood (47), *Enterococcus faecium* growth in human serum (48), and *Bacillus anthracis* growth in human serum and virulence in a mouse bacteremia model (42). Likewise, purine biosynthesis is required for *S. aureus* growth in bovine and human serum (49), virulence in mouse models of bacteremia (36, 38), and endocarditis infections (39). Therefore, it is not surprising that purine biosynthesis is also required for *E. faecalis* to establish a successful infection in wounds, especially during the early phase of wound infection where *E. faecalis* is replicating (11). Together, these studies demonstrate that *de novo* purine biosynthesis probably is a metabolic pathway that is important for many bacterial pathogens during infection. Hence, finding ways to locally sequester exogenous purines in the wound microenvironment may be useful in controlling *E. faecalis* wound infection and CAUTI.

Even though we showed that *de novo* purine biosynthesis is important for *E. faecalis* replication during acute wound infection, the levels of purine metabolites in the wound microenvironment remained similar between wild-type OG1RF- and OG1RF ∆*purEK*-infected (defective in purine biosynthesis) wounds (i.e., OG1RF ∆*purEK* mutant did not correlate with more purines at the wound microenvironment as we predicted). Apart from the *de novo* purine biosynthesis pathway, bacteria can recycle purines from nucleic acid (such as nucleotides, nucleosides, and nucleobases) from the external environment through the purine salvage pathway (34, 50). Therefore, we speculate that the purine salvage pathway may play a role in providing purines to *E. faecalis* (wild-type OG1RF and OG1RF ∆*purEK*). However, the added advantage of being able to perform *de novo* purine biosynthesis in wild-type OG1RF clearly contributes to its fitness during *in vivo* competitive infection and better colonization during single-strain wound infection compared to OG1RF ∆*purEK*. Since we did not observe significant fitness defects for mutants disrupted in the purine salvage pathway or significant gene expression changes in genes required for purine salvage pathway in our *in vivo* Tn-seq and RNA-seq at 8 hpi, respectively, the data suggest that *de novo* purine biosynthesis likely makes a greater contribution than purine salvage during acute *E. faecalis* wound infection.

Carbohydrates are essential for their metabolism into glucose, which serves as a primary energy source for most bacteria. These large uncharged polar molecules cannot cross the bacterial plasma membrane freely (51). Consequently, bacteria encode PTS to import carbohydrates from the environment (37). A PTS is made up of several functional subunits, of which the EII subunits of each PTS determines its substrate carbohydrate specificity (37). As such, most bacteria encode multiple PTS to enable the import of different carbohydrates. As the wound infection progresses, *E. faecalis* encounters changing carbohydrate availability in the wound microenvironment from higher glucose during acute wound infection to higher galactose and mannose in late stages of the infection, and because *E. faecalis* can persist and survive the change in carbohydrate availability, it hints that there is a metabolic switch in the carbohydrate phosphotransferase system in *E. faecalis* and that wound infection is a “controlled” process. As a result, any disturbance introduced (e.g., hindered mannose import) to this “controlled” process would then lead to reduced competitive index as observed with the OG1RF ∆*mptD* mutant during wound infection. However, there is still limited information on carbohydrate availability in wounds in other animal models or in human wounds; hence, we cannot discount the presence of other carbohydrates in the wound microenvironment and the importance of other PTS that might be contributing to *E. faecalis* persistence. Apart from *E. faecalis*, the impact of carbohydrate metabolism and import is also evident in the pathogenesis of several other Gram-positive bacteria. For example, sucrose-6-phosphate hydrolase and its sucrose ABC transporter contribute to *Streptococcus pneumoniae in vivo* fitness during lung infection in mouse (52). Based on comparative genomic analysis, a PTS locus in *Enterococcus faecium* clinical isolates is found to play an important role in mouse intestinal colonization, and the deletion of an EII subunit of this PTS resulted in reduced intestinal colonization (53). Garnett et al. (54) similarly showed the importance of *S. aureus* PTS in importing carbohydrates from the airway surface liquid to support its growth. Given the significance of PTS on the pathogenesis of various infections caused by different bacteria, drugs and/or inhibitors targeting carbohydrate import process(s) seem like an attractive alternative to control infections.

As aforementioned, purine biosynthesis may be a common metabolic pathway that is required for virulence. Fittingly, *E. faecalis de novo* purine biosynthesis also contributes to its fitness during CAUTI. The notion that purines are limiting in the urinary tract is consistent with studies of uropathogenic *E. coli*, in which a *guaA* mutant that has defective guanine biosynthesis was unable to grow in human urine *in vitro* and was significantly less virulent than the parental wild-type strain in a mouse model of UTI (43). The OG1RF ∆*mptD* mutant was less fit than wild type during CAUTI, suggesting that availability of galactose and mannose in the urinary tract is likely limited, which is in contrast with uropathogenic *E. coli* that preferentially take advantage of amino acids and small peptides as a carbon source, since mutants with defective peptide import had significantly reduced fitness during UTI (55). However, future studies will be needed to confirm whether purines are similarly limited as well as the carbohydrate profile in the mice bladders during CAUTI.

*E. faecalis de novo* purine biosynthesis and MptABCD PTS are functionally linked (Fig. 5B). There are multiple pathways like the pentose phosphate pathway; alanine, aspartate, and glutamate metabolism; thiamine metabolism; and histidine metabolism that contribute to purine biosynthesis (efi00230) (56), and it is possible that in order to maintain healthy levels of purine to support active cell division during *E. faecalis* acute replication, the presence of all the contributing pathways is likely required. This could explain why there is a growth attenuation of OG1RF ∆*mptD* mutant in purine lacking medium as purine biosynthesis is affected in the absence of mannose transport. However, the OG1RF ∆*mptD* mutant is outcompeted compared to OG1RF ∆*purEK* in wounds at 3 dpi, suggesting that the import of galactose and mannose likely has a more significant role as a carbon source for *E. faecalis* persistence in wounds other than for purine biosynthesis. A point to consider is that genes encoding fructose and mannose metabolism pathways as well as their PTS were downregulated in 8 hpi wounds. An explanation could be that during acute replication, cells are actively proliferating, and therefore, genes encoding purine metabolism, peptidoglycan, and fatty acid biosynthesis are upregulated. At the same time, it is likely that bacterial cells would still require some level of carbohydrates to be imported for their growth. Thus, it is possible that when *mptD* is deleted, this basic requirement of carbohydrates (galactose and mannose) might not be met, and therefore, we observed a decrease in OG1RF ∆*mptD* fitness compared to wild-type OG1RF at 8 hpi. These observations suggest that genes can have a functional shift depending on the state/phase during *E. faecalis* wound infection.

An outstanding question from this study is whether shikimate biosynthesis may be contributing to *E. faecalis* persistence in wounds. The production of secondary metabolites is usually not critical for cell growth but instead serves as a survival strategy for organisms during adverse conditions likely triggered by the depletion of nutrients or environmental stress (57). The end product of the shikimate pathway is chorismate, which is essential for subsequent biosynthesis of aromatic amino acids such as phenylalanine, tryptophan, and tyrosine as well as aromatic secondary metabolites (58, 59). For example, chorismate branching into the synthesis of para-aminobenzoic acid (PABA), which is a precursor for folate metabolism (59). Interestingly, Turner et al. (33) not only showed that purines were “not available” to *P. aeruginosa* during wound infection but also showed that chorismate, phenylalanine, tyrosine, and PABA were “not available.” Moreover, shikimate pathway intermediates are also potential substrates leading to other metabolic pathways (58). Thereby, it is tempting to hypothesize that the shikimate pathway is important as it is a central metabolic route that leads to the production of other aromatic metabolites that might be essential for *E. faecalis* persistence in wounds. However, further studies will be needed to examine the role of *E. faecalis* shikimate biosynthesis during wound infection.

**Fig 7:**
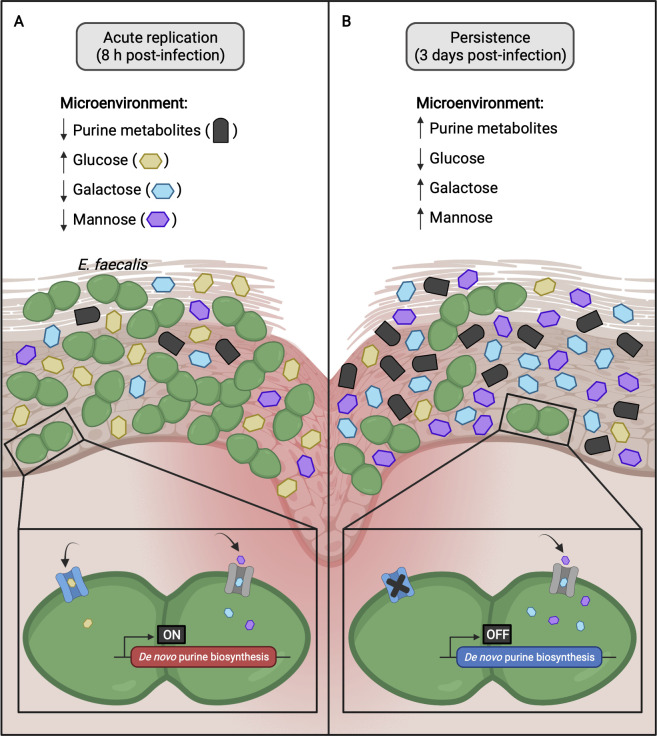
Proposed working model of *E. faecalis* wound infection dynamics. (**A**) During *E. faecalis* acute replication (8 h post-infection), purine metabolites and galactose and mannose availability in the wound microenvironment are low, while glucose is high. As a result, *de novo* purine biosynthesis is induced, and *E. faecalis* imports all three carbohydrates to support its growth. (**B**) However, as the wound infection progresses to persistence (3 days post-infection), purine metabolites and galactose and mannose availability are high, while glucose is low. Since *E. faecalis* is not actively dividing and purine metabolites are abundant in the wound microenvironment, *de novo* purine biosynthesis is likely not induced in *E. faecalis*. Additionally, given that there was minimal depletion of glucose and increased uptake of galactose and mannose by *E. faecalis*, it suggests that galactose and mannose are the preferred carbohydrates by *E. faecalis* during persistence. The figure was created with BioRender.com.

Based on our current findings, we propose a working model of *E. faecalis* wound infection dynamics (Fig. 7). During the early phase of wound infection, *E. faecalis* undergoes an acute replication, and therefore, the demand for purines is high. However, purine metabolites in the wound microenvironment are consumed by *E. faecalis*, which makes the *E. faecalis de novo* purine biosynthesis indispensable for the acute replication (Fig. 7A). Carbohydrate availability during acute infection differs from persistence, in which glucose is higher while galactose and mannose are lower during earlier stages of infection. Despite these differences in availability, it is likely that *E. faecalis* can use all three carbohydrates to support its growth. By contrast, during *E. faecalis* persistence in wounds, availability of galactose and mannose is higher than during acute replication and that there was minimal depletion of glucose by *E. faecalis*, suggesting that galactose and mannose are a preferred carbohydrate source by *E. faecalis* during persistence (Fig. 7B).

Overall, our study provides insights into the pathogenic requirements and potential of *E. faecalis* during wound infection and factors that are required for *E. faecalis* to replicate and persist in this niche. Given the suggested importance of *E. faecalis de novo* purine biosynthesis and MptABCD PTS during acute replication and persistence in wounds, this work raises the possibility for future drugs and/or inhibitors to sequester exogenous purines in the wound microenvironment or to target MptABCD PTS or, in general, *E. faecalis* carbohydrate utilization processes as a novel approach to curb infections.

## Data Availability

All the sequences have been deposited in the National Center for Biotechnology Information (NCBI) Gene Expression Omnibus (GEO) database under accession number GSE206751.

## SUPPLEMENTAL MATERIAL

File S1 mbio.02384-23-s0001.xlsx Complete tables of Tn-seq and RNA-seq data analysis.

Figure S1 mbio.02384-23-s0002.pdf Transposon insertions in *E. faecalis de novo* purine biosynthesis genes are among the most significantly underrepresented genes at 8 hpi.

Figure S2 mbio.02384-23-s0003.pdf *E. faecalis* pathways that are significantly enriched in 8 hpi wounds.

Figure S3 mbio.02384-23-s0004.eps No significant differences between adenosine, guanosine, and inosine metabolite levels during *E. faecalis* wound infection.

Figure S4 mbio.02384-23-s0005.pdf Transposon insertions in *mptABCD* are among the most significantly underrepresented genes at 3 dpi.

Figure S5 mbio.02384-23-s0006.eps MptABCD phosphotransferase system contributes to *E. faecalis* wound fitness during persistence in diabetic mice.

Legends mbio.02384-23-s0007.docx Supplemental legends.

Table S1 mbio.02384-23-s0008.docx Bacterial strains used in this study.

Table S2 mbio.02384-23-s0009.docx Primers used in this study.

Table S3 mbio.02384-23-s0010.docx Complete table for carbohydrate fermentation test (API 50 CH) of wild-type OG1RF and mutant strains.

ASM does not own the copyrights to Supplemental Material that may be linked to, or accessed through, an article. The authors have granted ASM a non-exclusive, world-wide license to publish the Supplemental Material files. Please contact the corresponding author directly for reuse.
