# Supplementary figures and images for: Purine and carbohydrate availability drive Enterococcus faecalis fitness during wound and urinary tract infections
Source: mBio. 2023 Dec 11;15(1):e02384-23. doi: 10.1128/mbio.02384-23 (PMC10790769; doi:10.1128/mbio.02384-23)

**A**

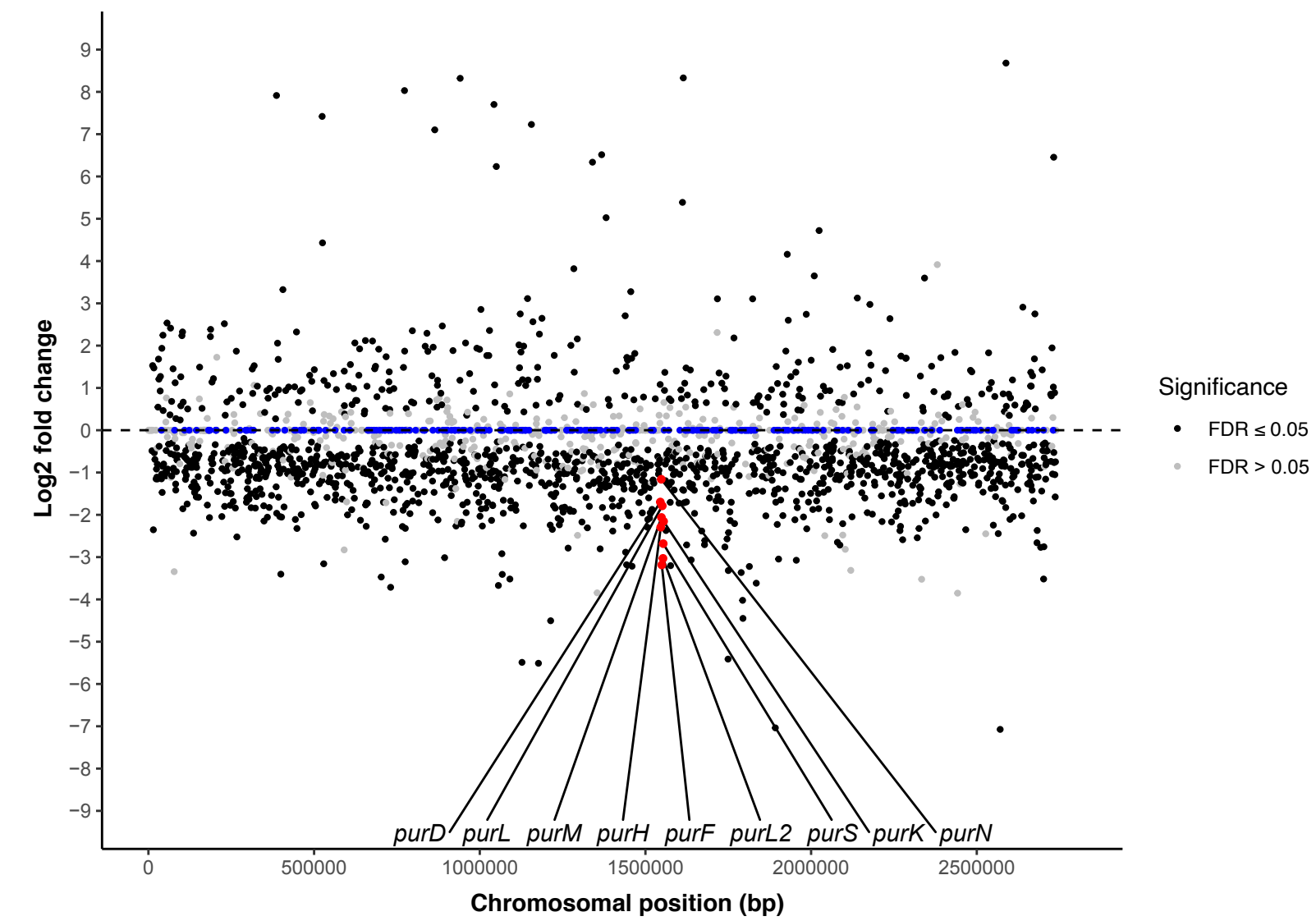

**B**

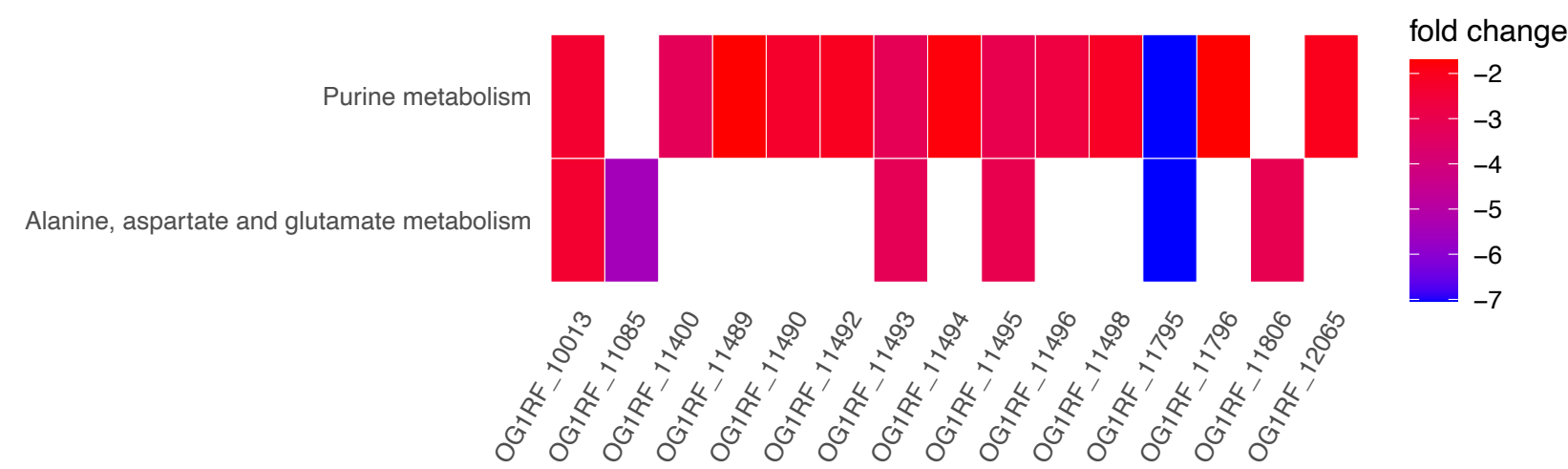

**C**

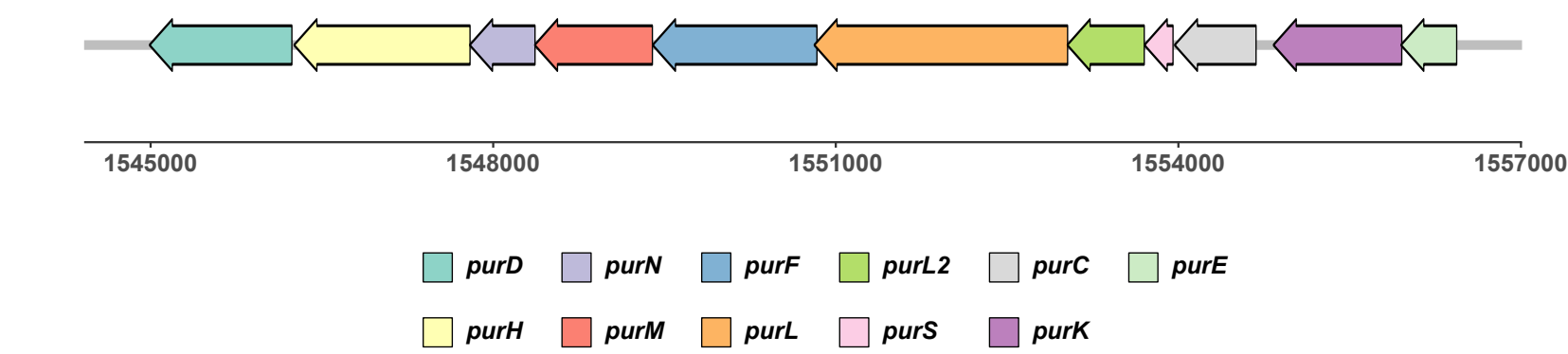

**D**

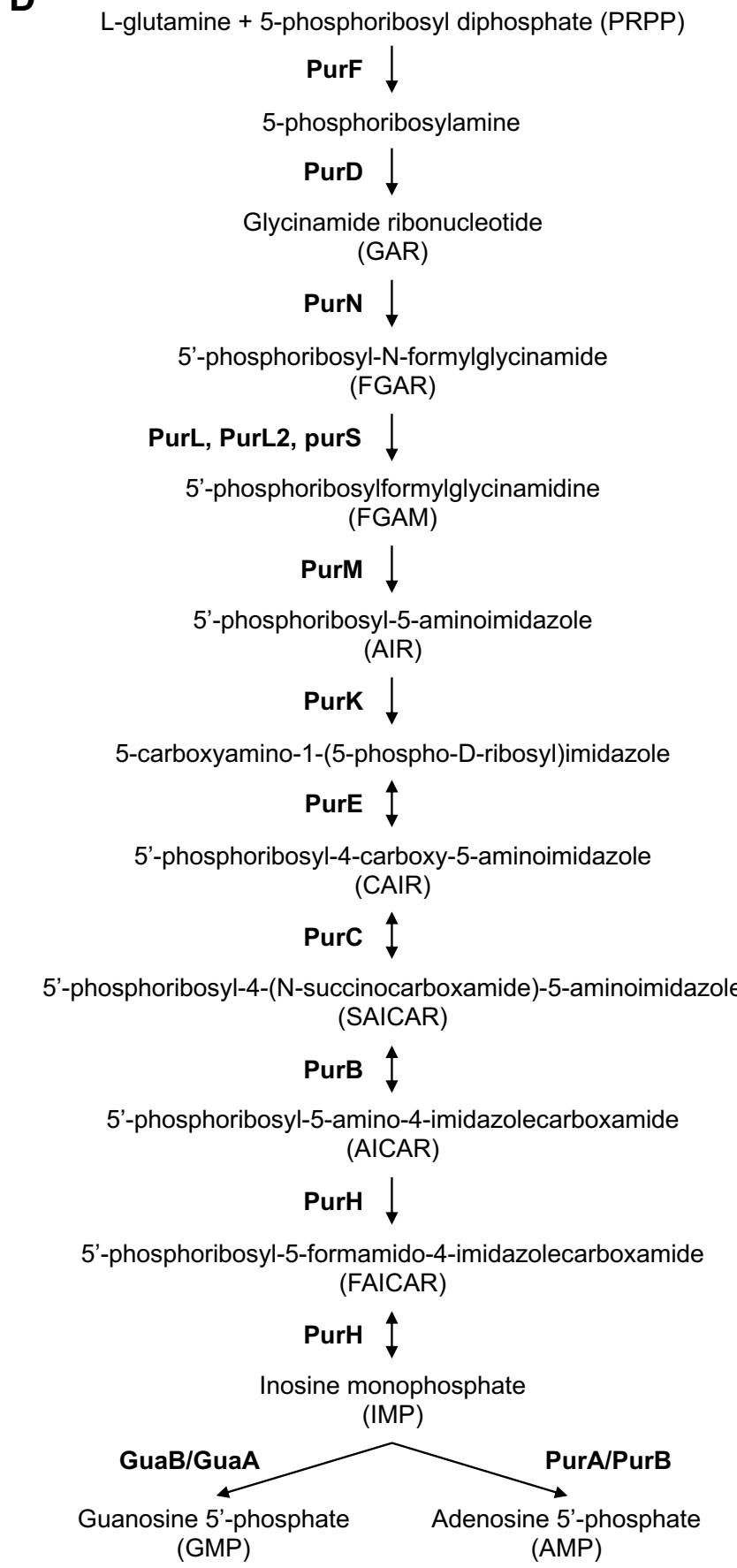

Supplement: Figure S1 — Transposon insertions in E. faecalis de novo purine biosynthesis genes are among the most significantly underrepresented genes at 8 hpi. [file mbio.02384-23-s0002.pdf]

**A**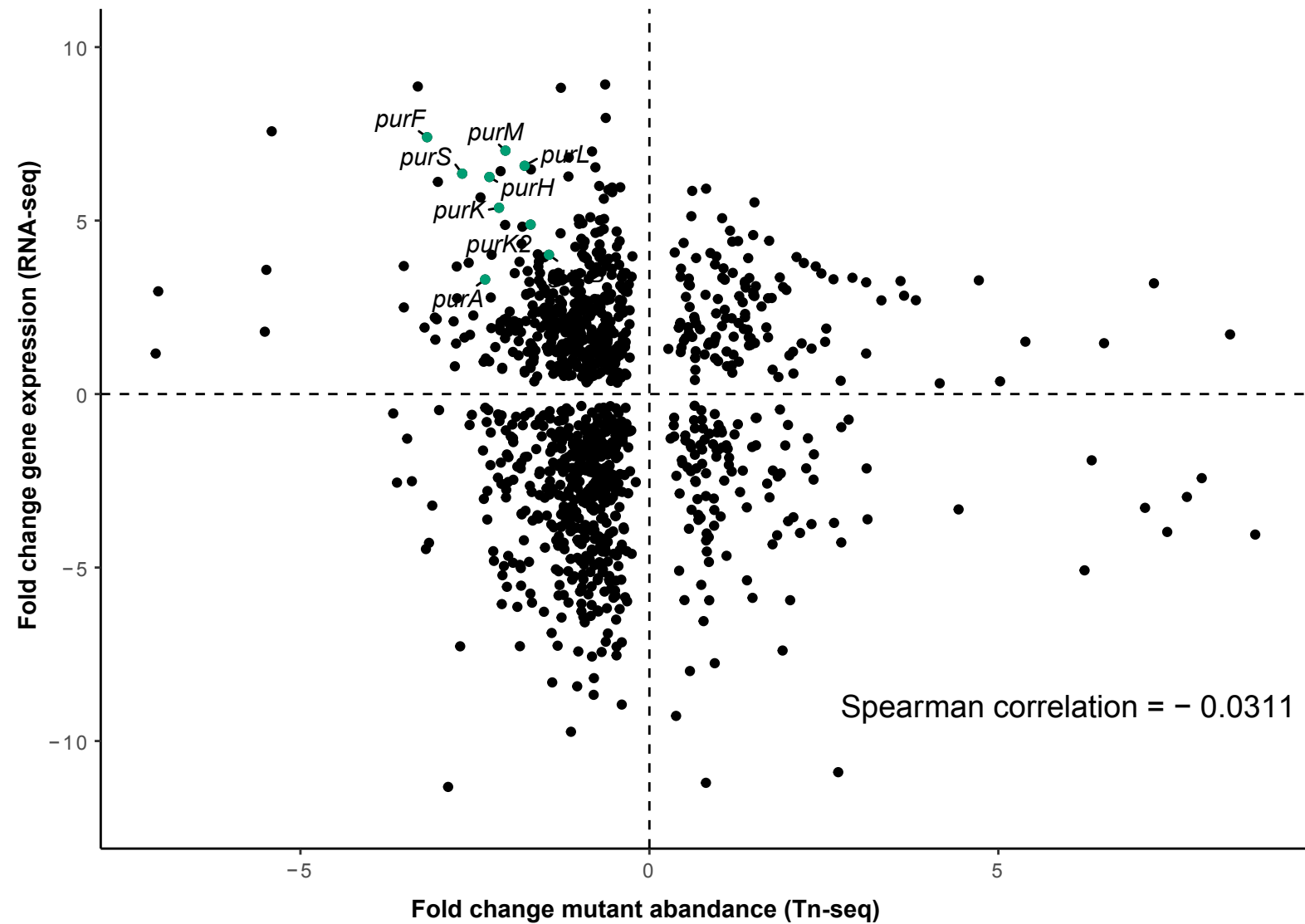**B**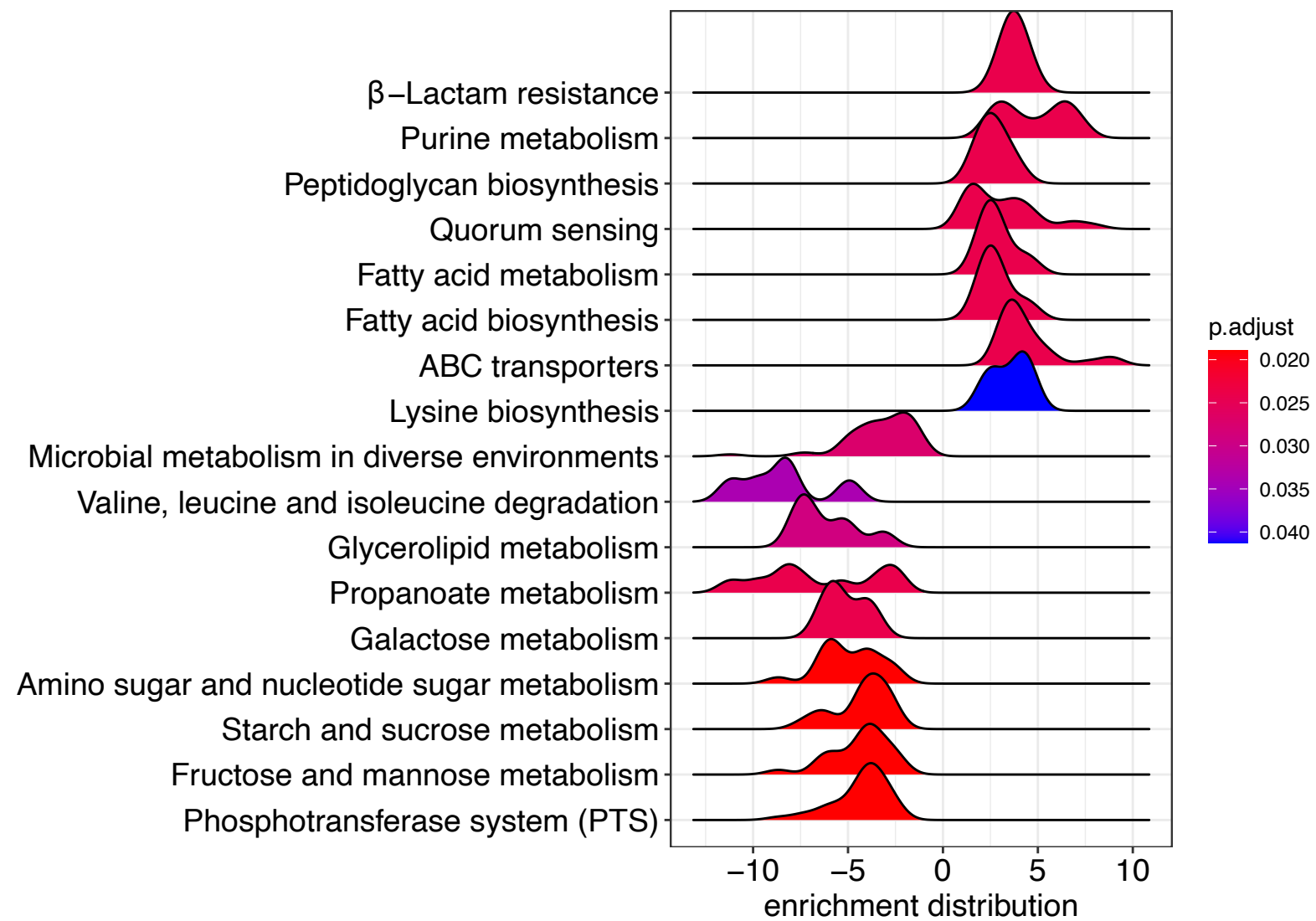

Supplement: Figure S2 — E. faecalis pathways that are significantly enriched in 8 hpi wounds. [file mbio.02384-23-s0003.pdf]

**A**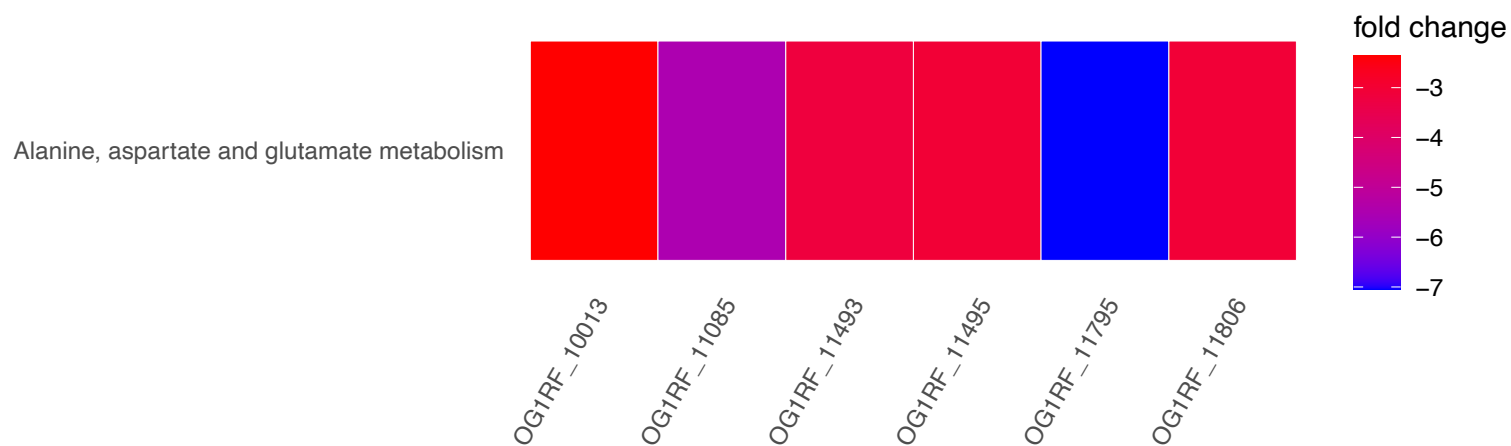**B**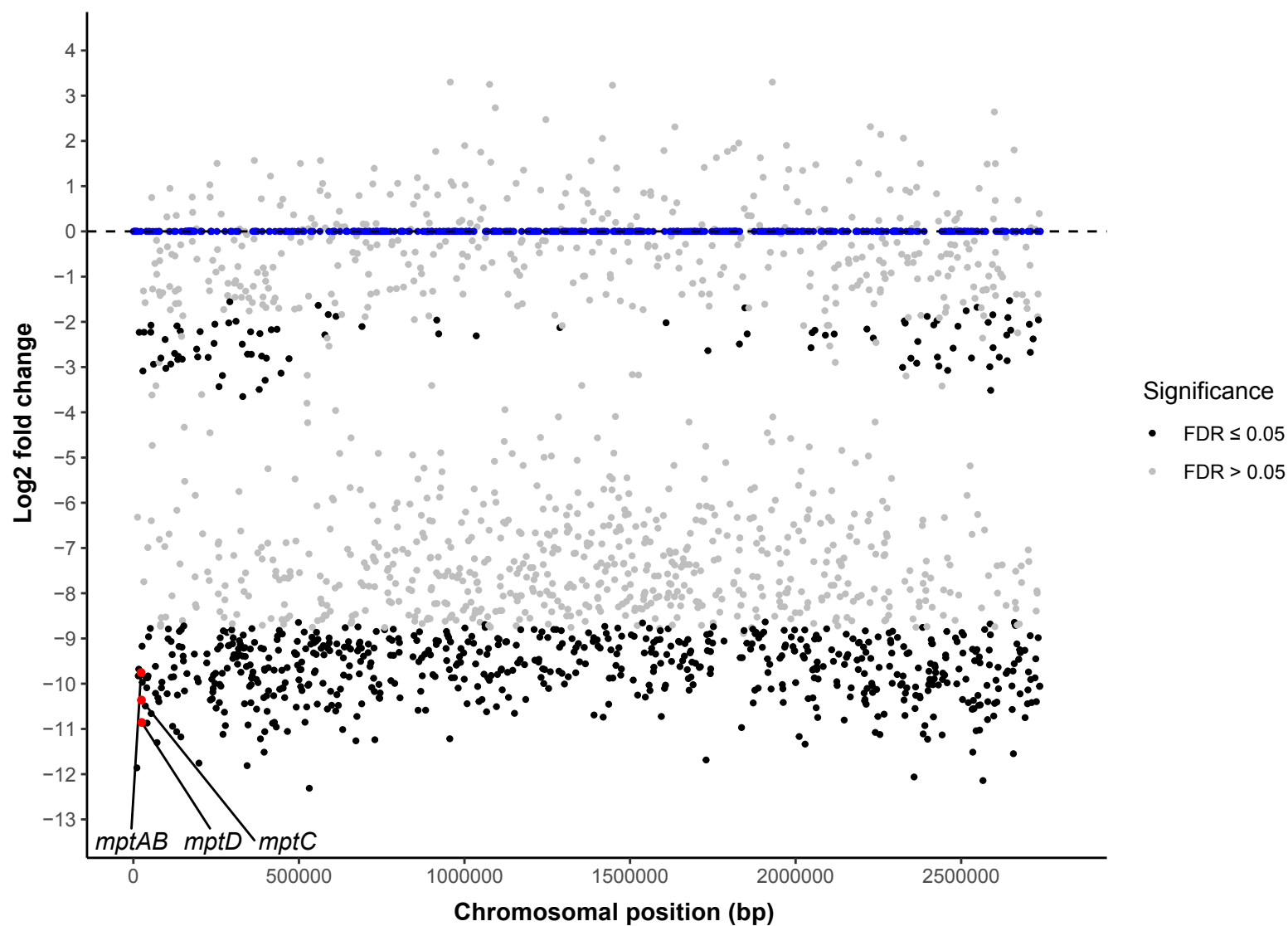

Supplement: Figure S4 — Transposon insertions in mptABCD are among the most significantly underrepresented genes at 3 dpi. [file mbio.02384-23-s0005.pdf]
