# Supplementary material for: Purine and carbohydrate availability drive Enterococcus faecalis fitness during wound and urinary tract infections
Source: mBio. 2023 Dec 11;15(1):e02384-23. doi: 10.1128/mbio.02384-23 (PMC10790769; doi:10.1128/mbio.02384-23)
Supplement: Legends — Supplemental legends. [file mbio.02384-23-s0007.docx]

**Supplementary Figure 1 Transposon insertions in *E. faecalis de novo* purine biosynthesis genes are among the most significantly underrepresented genes at 8 hpi. (A)** Distribution of *E. faecalis* transposon mutant abundance profiled by Tn-seq from 8 hpi wounds. Significant mutants from Tn-seq analysis are colored black (p ≤ 0.05 and FDR ≤ 0.05). *E. faecalis* genes with no transposon mutant found in the transposon library are colored in blue. **(B)** Gene set enrichment pathways identified based on significantly underrepresented genes identified from Tn-seq analysis at 8 hpi. **(C)** Operon arrangement of purine biosynthesis genes and **(D)** purine biosynthesis pathway in *E. faecalis*. Adapted from KEGG pathways efi00230.

**Supplementary Figure 2 *E. faecalis* pathways that are significantly enriched in 8 hpi wounds. (A)** Correlation plot of statistically significant genes from Tn-seq and RNA-seq. **(B)** Ridge plot showing the distribution of fold-change for genes in significantly enriched pathways identified from GSEA based on significantly differentially expressed genes between *E. faecalis* wild-type OG1RF inoculum and wild-type OG1RF-infected wounds harvested at 8 hpi. Color gradient represents false discovery rate (p.adjust values).

**Supplementary Figure 3 No significant differences between adenosine, guanosine, and inosine metabolite levels during *E. faecalis* wound infection.** Male C57BL/6 mice were wounded and inoculated with PBS or 2 – 4 × 10^6^ CFU of wild-type OG1RF. Wounds were harvested at 8 hpi and 3 dpi for quantification of **(A)** adenosine, **(B)** guanosine, and **(C)** inosine using LC-MS. Each data point represents one mouse and error bars represent SD from the mean; N = 2, n = 5 mice per group per experiment. Statistical analysis was performed using the Mann-Whitney U test, *p < 0.05, **p < 0.01, ***p < 0.001. Male C57BL/6 mice were wounded and inoculated with PBS or 2 – 4 × 10^6^ CFU of wild-type OG1RF or OG1RF ∆*purEK*. Wounds were harvested at 8 hpi for quantification of **(D)** adenine, **(E)** xanthine, **(F)** adenosine, **(G)** guanosine, and **(H)** inosine using LC-MS. Each data point represents one mouse and error bars represent SD from the mean; N = 2, n = 5 mice per group per experiment. Statistical analysis was performed using the Mann-Whitney U test, *p < 0.05, **p < 0.01, ***p < 0.001.

**Supplementary Figure 4 Transposon insertions in *mptABCD* are among the most significantly underrepresented genes at 3 dpi. (A)** Gene set enrichment pathways identified based on significantly underrepresented genes identified from Tn-seq analysis at 3 dpi. **(B)** Distribution of *E. faecalis* transposon mutant abundance profiled by Tn-seq from 3 dpi wounds. Significant mutants from Tn-seq analysis are colored black (p ≤ 0.05 and FDR ≤ 0.05). *E. faecalis* genes with no transposon mutant found in the transposon library are colored in blue.

**Supplementary Figure 5 MptABCD phosphotransferase system contributes to *E. faecalis* wound fitness during persistence in diabetic mice.** Growth kinetics of wild-type OG1RF pMPSP3535::P*_nisA_*-Empty, OG1RF ∆*mptD* pMSP3535::P*_nisA_*-Empty, and OG1RF ∆*mptD* pMSP3535::*P_nisA_-mptD* in M9Y media supplemented **(A)** without additional carbohydrates and with 1% (w/v) **(B)** galactose, **(C)** mannose, and **(D)** glucose over 16 h. Plasmid-based *mptD* expression was induced with 40 ng/mL nisin. Baseline readings are indicated by Blank, containing only the growth media. Data are mean values of three independent biological replicates and vertical lines represent SD from the mean. Statistical analysis was performed at 16 h OD_600_ measurement with wild-type OG1RF pMPSP3535::P*_nisA_*-Empty as the comparator using the Mann-Whitney U test, **p < 0.01, ****p < 0.0001. **(E)** Male and female *db/db* mice were wounded and infected with a 1:1 ratio of *E. faecalis* OG1X:wild-type OG1RF or OG1X:OG1RF ∆*mptD* at 2 – 4 × 10^6^ CFU/wound (N = 4, n = 4 – 5 mice) and CFU determined at 3 dpi. The recovered bacteria were enumerated on selective agar plates for each strain. Each data point represents one mouse and horizontal lines indicate the median. Data points in red and black represents 7 – 8 weeks and 14 weeks old mice, respectively. Statistical analysis was performed using the Mann-Whitney U test, ****p < 0.0001.

**Supplementary file 1 (Sheet 1)** Complete table of *E. faecalis* transposon mutant abundance profiled by Tn-seq from 8 hpi wounds. **(Sheet 2)** Complete table of *E. faecalis* differentially expressed genes from 8 hpi wounds. **(Sheet 3)** Accessory genes of *E. faecalis* wild-type OG1RF and OG1X identified from whole genome comparison. **(Sheet 4)** Complete table of *E. faecalis* transposon mutant abundance profiled by Tn-seq from 3 dpi wounds. **(Sheet 5)** Complete table of differentially expressed genes in *E. faecalis* wild-type OG1RF relative to OG1RF ∆*mptD* when grown in TSBd supplemented with 1% (w/v) galactose. **(Sheet 6)** Complete table of unique differentially expressed genes in *E. faecalis* OG1RF ∆*mptD* relative to wild-type OG1RF when grown in TSBd supplemented with 1% (w/v) mannose. **(Sheet 7)** Complete table of unique differentially expressed genes in *E. faecalis* wild-type OG1RF relative to OG1RF ∆*mptD* when grown in TSBd supplemented with 1% (w/v) mannose. **(Sheet 8)** Complete table of differentially expressed genes in *E. faecalis* wild-type OG1RF relative to OG1RF ∆*mptD* when grown in TSBd without mannose or galactose supplementation. **(Sheet 9)** Complete table of differentially expressed genes in *E. faecalis* wild-type OG1RF relative to OG1RF ∆*mptD* when grown in TSBd supplemented with 1% (w/v) mannose. **(Sheet 10)** Complete table of common *E. faecalis* differentially expressed genes found in **Sheet 8** and **Sheet 9**. Common differentially expressed genes identified in **Sheet 10** were removed from **Sheet 9**, giving rise to unique differentially expressed genes in **Sheet 7**.
