# Supplementary material for: Purine and carbohydrate availability drive Enterococcus faecalis fitness during wound and urinary tract infections
Source: mBio. 2023 Dec 11;15(1):e02384-23. doi: 10.1128/mbio.02384-23 (PMC10790769; doi:10.1128/mbio.02384-23)
Supplement: Table S1 — Bacterial strains used in this study. [file mbio.02384-23-s0008.docx]

**Supplementary Table 1 Bacterial strains used in this study.**

| **Strain** | **Description** | **Reference** |
| --- | --- | --- |
| OG1X | Wild-type, str^r^ | [1] |
| OG1RF | Wild-type, rif^r^ | [2] |
| OG1RF pTCV::P*_tet_*-Empty | OG1RF with pTCV empty vector, erm^r^, kan^r^ | This study |
| OG1RF pMSP3535::P*_nisA_*-Empty | OG1RF with pMSP3535 empty vector, erm^r^ | This study |
| OG1RF ∆*purEK* | OG1RF with *purE* and *purK* deletion | This study |
| OG1RF ∆*purEK* pTCV::P*_tet_*-Empty | ∆*purEK* with pTCV empty vector, erm^r^, kan^r^ | This study |
| OG1RF ∆*purEK* pTCV::P*_tet_*-*purEK* | ∆*purEK* with *purEK* complemented on pTCV vector, erm^r^, kan^r^ | This study |
| OG1RF ∆*mptD* | OG1RF with *mptD* deletion | This study |
| OG1RF ∆*mptD* pMSP3535::P*_nisA_*-Empty | ∆*mptD* with pMSP3535 empty vector, erm^r^ | This study |
| OG1RF ∆*mptD* pMSP3535::P*_nisA_*-*mptD* | ∆*mptD* with *mptD* complemented on pMSP3535 vector, erm^r^ | This study |

*Str^r^, rif^r^, erm^r^ and kan^r^ represents streptomycin, rifampicin, erythromycin, and kanamycin resistance, respectively.*

**REFERENCES**

1. Ike, Y., Craig, R.A., White, B.A., Yagi, Y., and Clewell, D.B. Modification of *Streptococcus faecalis* sex pheromones after acquisition of plasmid DNA*.* *Proceedings of the National Academy of Sciences*. 1983. 80(17): 5369-73.

2. Dunny, G.M., Brown, B.L., and Clewell, D.B. Induced cell aggregation and mating in *Streptococcus faecalis*: evidence for a bacterial sex pheromone*.* *Proceedings of the National Academy of Sciences*. 1978. 75(7): 3479-3483.
