# Supplementary material for: Purine and carbohydrate availability drive Enterococcus faecalis fitness during wound and urinary tract infections
Source: mBio. 2023 Dec 11;15(1):e02384-23. doi: 10.1128/mbio.02384-23 (PMC10790769; doi:10.1128/mbio.02384-23)
Supplement: Table S2 — Primers used in this study. [file mbio.02384-23-s0009.docx]

**Supplementary Table 2 Primers used in this study.**

| **Purpose** | **Primer name** | **Sequence (5' to 3')** | **Reference** |
| --- | --- | --- | --- |
| Construction of OG1RF ∆*purEK* | 1-∆*purEK*_UpF | CATGCTCGAGCGGCCGTGTAGAGGTTATCGAAACACGG | This study |
|  | 2-∆*purEK*_UpR | GTTGCTCCGACTTTTAAAGGTTTACATGAATGTAATAACTCCTTCCG | This study |
|  | 3-∆*purEK*_DownF | CGGAAGGAGTTATTACATTCATGTAAACCTTTAAAAGTCGGAGCAAC | This study |
|  | 4-∆*purEK*_DownR | TACCGAGCTCGGATCTGTCCTTTTCCATCGTATCCACC | This study |
|  | screen-∆*purEK_*F | GAACAGATCGAAGAAGGGCA | This study |
|  | screen-∆*purEK_R* | AGGGGCAATCTTACAGCCAA | This study |
| Construction of OG1RF ∆*mptD* | 1-∆*mptD*_UpF | CATGCTCGAGCGGCC GTGTTAAAGGTGTTCCTTCAGCGA | This study |
|  | 2-∆*mptD*_UpR | AAGCCGATTAAGTGGCCGACTGCCATTTTCTTTGCTCCTCC | This study |
|  | 3-∆*mptD*_DownF | GGAGGAGCAAAGAAAATGGCAGTCGGCCACTTAATCGGCTT | This study |
|  | 4-∆*mptD*_DownR | TACCGAGCTCGGATC CCCAATCGAGAGAATCCCTCT | This study |
|  | screen-∆*mptD_*F | GTATGGTCGTTGCCGTAGGT | This study |
|  | screen-∆*mptD_R* | TCCTGTAAATGCCGTCGCA | This study |
| Construction of OG1RF ∆*purEK* pTCV::P*_tet_*-*purEK* | *purEK*compl_F | CTGAGGATCCCAGTGAAAAAAGGCGGAAGGAGT | This study |
|  | *purEK*compl_R | ACTGGCATGCCTTGCTTGGAGCAAGCATTTATTAAG | This study |
|  | screen-*purEK*compl_F | GTAAAACGACGGCCAGT | This study |
|  | screen-*purEK*compl_R | CAGGAAACAGCTATGAC | This study |
| Linearizing pMSP3535 | iPCR_pMSP3535_F | GATCCGGTACCACTAGTCCCG | This study |
|  | iPCR_pMSP3535_R | CATGCAGAGTCTCCTGTTTTACAAC | This study |
| Construction of OG1RF ∆*mptD* pMSP3535::P*_nisA_*-*mptD* | *mptD*compl_F | AGGAGACTCTGCATGCTAATTCTGAAGGAGGAGCAAA | This study |
|  | *mptD*compl_R | TAGTGGTACCGGATCGACTAGTTTATAATAAGCCGATT | This study |
|  | screen-*mptD*compl_F | GGTTGCAAATTTTGAAAACCGC | This study |
|  | screen-*mptD*compl_R | TAATACGACTCACTATAGGG | This study |
