# Supplementary material for: Purine and carbohydrate availability drive Enterococcus faecalis fitness during wound and urinary tract infections
Source: mBio. 2023 Dec 11;15(1):e02384-23. doi: 10.1128/mbio.02384-23 (PMC10790769; doi:10.1128/mbio.02384-23)
Supplement: Table S3 — Complete table for carbohydrate fermentation test (API 50 CH) of wild-type OG1RF and mutant strains. [file mbio.02384-23-s0010.docx]

**Supplementary Table 3 Complete table for carbohydrate fermentation test (API 50 CH) of wild-type OG1RF pMPSP3535::P*_nisA_*-Empty, OG1RF ∆*mptD* pMSP3535::P*_nisA_*-Empty, and OG1RF ∆*mptD* pMSP3535::*P_nisA_-mptD*.**
